# Current and emerging tools for simultaneous assessment of infection and rejection risk in transplantation

**DOI:** 10.3389/fimmu.2024.1490472

**Published:** 2024-11-26

**Authors:** Dhakshayini Tharmaraj, William R. Mulley, Claire Dendle

**Affiliations:** ^1^ Department of Nephrology, Monash Health, Clayton, VIC, Australia; ^2^ Centre for Inflammatory Diseases, Department of Medicine, Monash University, Clayton, VIC, Australia; ^3^ Monash Infectious Diseases, Monash Health, Clayton, VIC, Australia

**Keywords:** infection, rejection, transplant, immunity, biomarkers, non-invasive, molecular diagnostics, dd-cfDNA

## Abstract

Infection and rejection are major complications that impact transplant longevity and recipient survival. Balancing their risks is a significant challenge for clinicians. Current strategies aimed at interrogating the degree of immune deficiency or activation and their attendant risks of infection and rejection are imprecise. These include immune (cell counts, function and subsets, immunoglobulin levels) and non-immune (drug levels, viral loads) markers. The shared risk factors between infection and rejection and the bidirectional and intricate relationship between both entities further complicate transplant recipient care and decision-making. Understanding the dynamic changes in the underlying net state of immunity and the overall risk of both complications in parallel is key to optimizing outcomes. The allograft biopsy is the current gold standard for the diagnosis of rejection but is associated with inherent risks that warrant careful consideration. Several biomarkers, in particular, donor derived cell-free-DNA and urinary chemokines (CXCL9 and CXCL10), show significant promise in improving subclinical and clinical rejection risk prediction, which may reduce the need for allograft biopsies in some situations. Integrating conventional and emerging risk assessment tools can help stratify the individual’s short- and longer-term infection and rejection risks in parallel. Individuals identified as having a low risk of rejection may tolerate immunosuppression wean to reduce medication-related toxicity. Serial monitoring following immunosuppression reduction or escalation with minimally invasive tools can help mitigate infection and rejection risks and allow for timely diagnosis and treatment of these complications, ultimately improving allograft and patient outcomes.

## 1 Introduction

With the increase in the global burden of chronic disease and the continued shortfall of transplantable organs, optimizing transplant recipient and allograft outcomes becomes paramount. Achieving and maintaining transplant tolerance, the ultimate goal of transplantation, requires an understanding of the individuals’ net state of immunity and achieving the optimal net-immune balance. Whilst over-immunosuppression is thought to lead to infective complications and underimmunosuppression to allograft rejection, the relationship between the two entities is likely more complicated and interlinked. Despite steady improvements in early allograft and patient survival, rejection, and infection continue to pose significant long-term risks (1). Balancing these two important complications remains a significant challenge for transplant clinicians.

An individual’s net state of immunity is a composite of their net state of immunosuppression and its resultant risk of infection and the net state of immune activation and its attendant risk of infection. The net state of immunity varies over time and is modulated by several factors, chiefly the degree of immunosuppression. Clinical risk assessments performed by Infectious Diseases and Transplant physicians may focus on infection and rejection risks in isolation, whereby optimal transplant recipient care and outcomes require the understanding of these risks in parallel. The clinical risk assessments are often further complicated by shared risk factors for both complications.

Strides to prevent and treat allograft rejection through potent immunosuppression increase susceptibility to infections. Conversely, infections can promote rejection by triggering immune mechanisms (e.g., heterologous immunity/alloreactive virus-specific T-cell activation, upregulation of surface markers and altered MHC class II signaling, *de novo* donor-specific antibody (DSA) formation and direct inflammation) or following the intentional reduction of immunosuppression to facilitate recovery from severe infection (2, 3). Furthermore, severe infections and the use of antimicrobial agents may affect immunosuppression medication levels through altered pharmacokinetic and pharmacodynamic profiles (4). Viruses pose a specific challenge, given their immune-evasive capabilities allowing for viral persistence and latency. Additionally, viral control relies on robust T-cell immune surveillance and responses, which are dampened by efforts to curb the risk of rejection.

Similarities in immune responses and clinical and histological features of infection and rejection, as is the case with polyomavirus (BK) nephropathy, can complicate therapeutic decision-making (5). Additionally, both rejection and infection episodes (particularly cytomegalovirus (CMV)) in the early post-transplant period predispose the recipient to both rejection and infective complications later on (2, 6–9). Immunosuppression reduction in response to leukopenia, a complication of both cytomegalovirus (CMV) infection and its’ treatments, has been associated with allograft rejection (10, 11). Moreover, lymphopenia/neutropenia, a well-described risk factor for primary and recurrent CMV disease, is a side effect of both immunosuppression (e.g., mycophenolate mofetil (MMF)) used to prevent rejection, and chemoprophylaxis used to prevent and treat infections such as CMV (e.g., valganciclovir, ganciclovir) and *Pneumocystis jirovecii* (PJP) (trimethoprim/sulfamethoxazole) (10, 11). Finally, rejection and infection can occur concurrently, rendering treatment options particularly difficult.

Current long-term monitoring of transplant health includes non-invasive and invasive measures. The conventional blood and urine parameters, serum creatinine, estimated glomerular filtration rate (eGFR), and proteinuria are not sensitive nor specific for rejection and often lag behind the onset of histological changes that may potentially be irreversible. Allograft biopsy, the current gold standard diagnostic test for rejection, is invasive, making it impractical for the regular surveillance of allograft health. It is also subject to large variabilities in sample adequacy and pathologists’ scoring (12, 13).

Emerging biomarkers show great promise in complementing conventional measures to improve their predictive power and pave the way for personalized transplant care and improved graft and patient survival. Detection of graft injury prior to changes in conventional markers may allow for early definitive histological diagnosis of rejection and timely therapy initiation prior to irreversible histological damage. Biomarkers may also provide valuable insight into the degree of immune activation and guide immunosuppression weaning strategies to reduce infection and toxicities. This review aims to describe the currently available and evolving tools relevant to simultaneously assessing the risk of infection and rejection. Informed, parallel infection and rejection risk assessments can allow clinicians to appropriately counsel recipients on their risks and personalize decisions around immunosuppression optimization and follow-up care.

## 2 Net state of immunity

A transplant recipient’s net state of immunity is dynamic and influenced by several host, donor, graft, surgical, immunosuppression, immunological, epidemiological, and environmental factors (14).

A shift towards an overall state of immune deficiency or activation increases the overall infection and rejection risks, respectively. Infection risk is primarily modulated by the degree of immune deficiency, epidemiological exposures, and preventative measures. Rejection risk increases with a state of immune activation, predisposed by an immunological mismatch/sensitization and influenced by inadequate immunosuppression levels.

**Figure 1:**
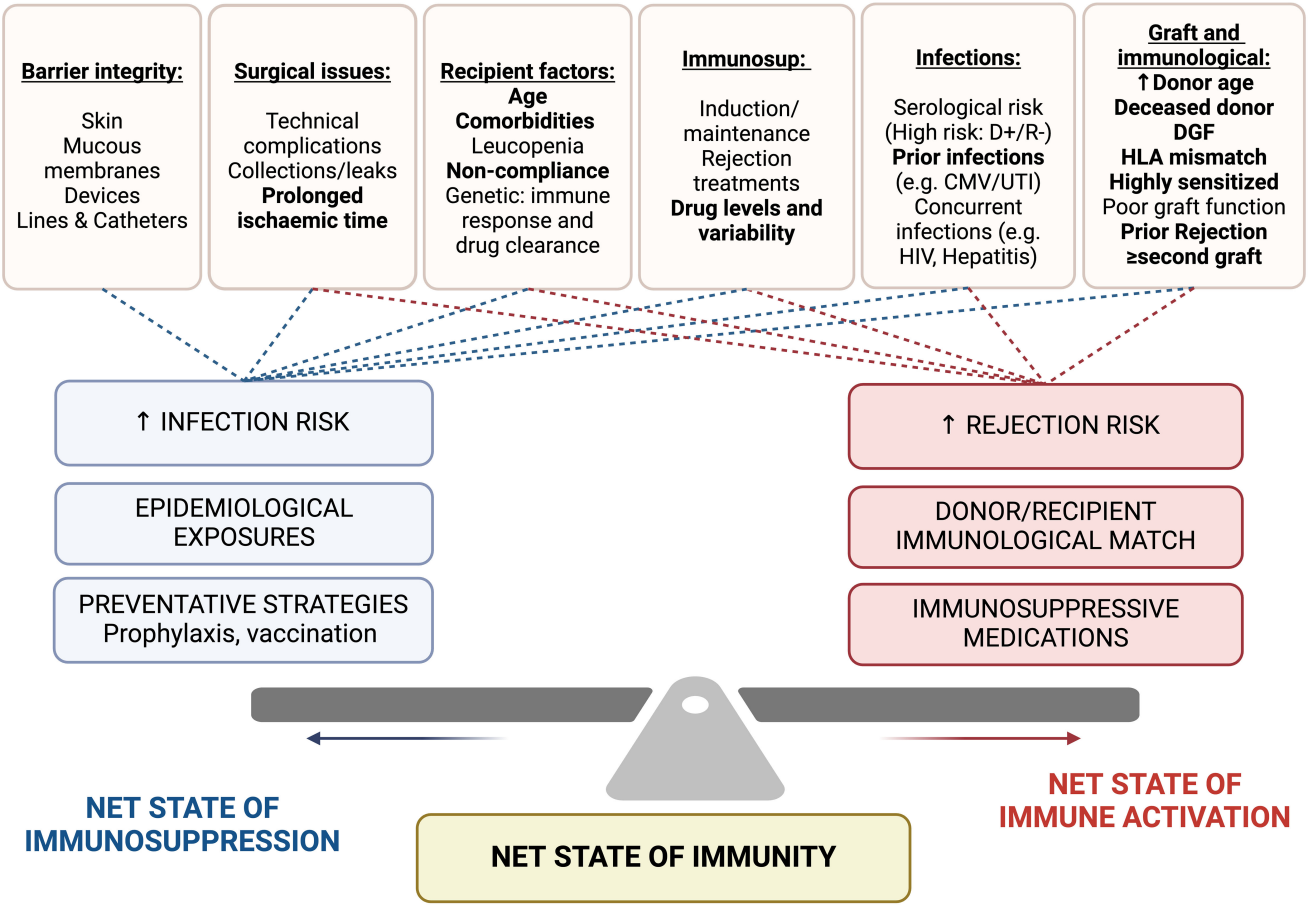
Factors impacting net state of immunosuppression (infection risk) and net state of immune activation (rejection risk). Shared risk factors are highlight in bold. Immunosup, immunosuppression; DGF, delayed graft function; HLA, human leukocyte antigen; D+/R-, Donor positive/Recipient negative; CMV, Cytomegalovirus; UTI, urinary tract infection; HIV, Human immunodeficiency virus.

Several shared risk factors for infection and rejection further complicate risk assessment strategies (**Figure 1**). These include a high degree of immunological mismatch (HLA mismatch and degree of sensitization), deceased donor transplantation, extended criteria donors, older donor age, prolonged cold ischemic time, delayed graft function, and prior history of rejection and infection (15–18).

An infection-rejection risk stratification model proposed by Cippa et al. (2015) revealed that older recipient age, deceased donor transplants, a higher number of HLA mismatches, and CMV donor +ve/recipient -ve status were highly associated with infection, while rejection was associated with deceased donor transplants, a higher number of HLA mismatches, and cyclosporin based immunosuppression (compared to tacrolimus) (19).

## 3 Cause-and-effect relationship between infection and rejection

**Figure 2:**
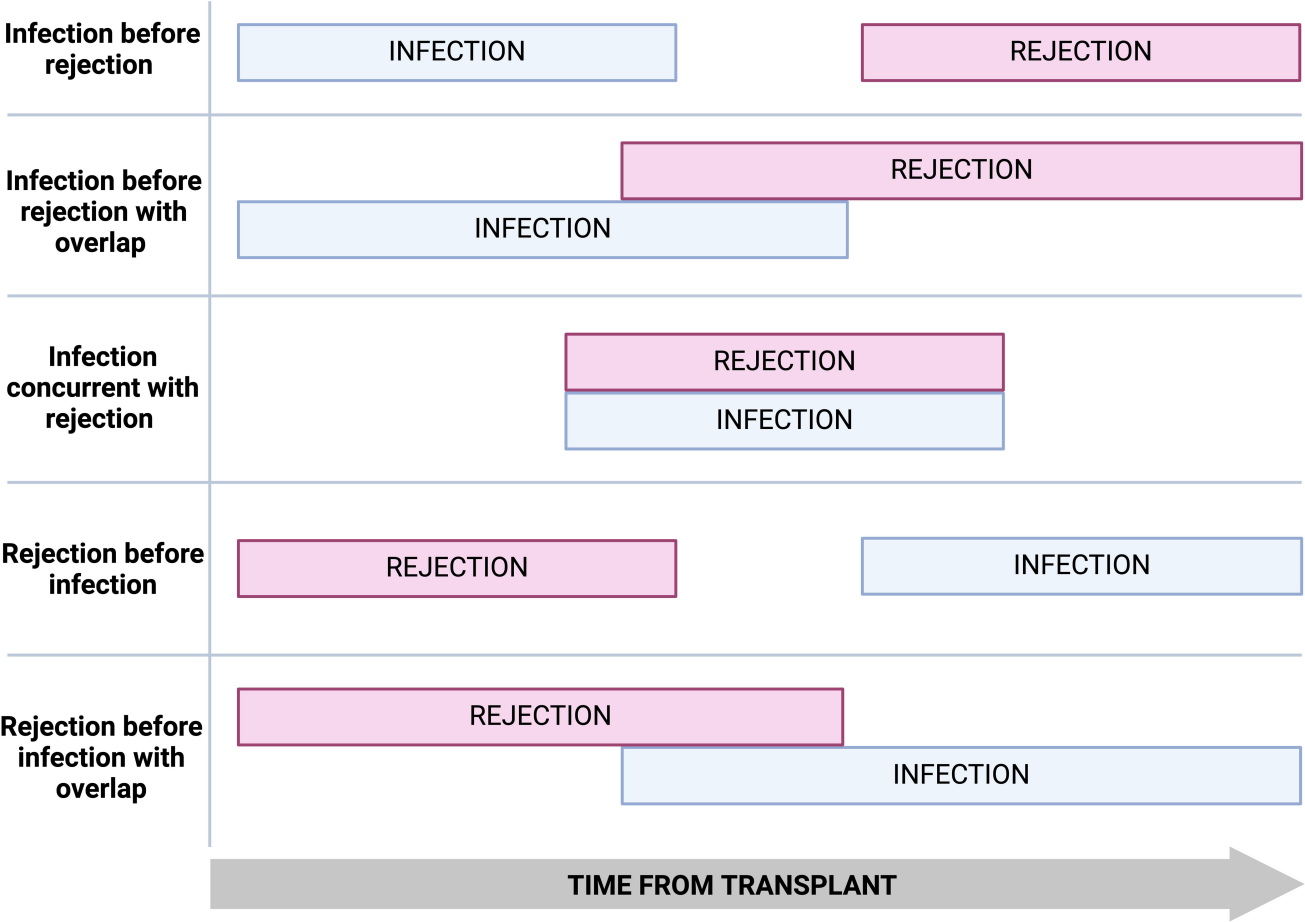
The possible temporal relationships between infection and rejection.

Significant temporal and geographical variations exist in infection and rejection rates post-transplantation. Infection and rejection episodes can occur in isolation, sequentially, with a period of overlap, or concurrently (**Figure 2**).

### 3.1 Infection causing rejection

Several pathogen-induced innate and adaptive immune responses can foster allosensitization and trigger an immune cascade that may lead to allograft rejection (2, 9). Direct graft inflammation, ischemia-reperfusion injury (IRI) following septic shock, priming of the adaptive immune response, T-cell phenotype switching from regulatory to inflammatory T-cells, heterologous immunity/cross-reactivity of virus-specific memory T-cells, and modulation of surface proteins (ICAM, VCAM) to facilitate immune cell infiltration or altering antigen (MHC-Class II) expression are postulated pathogen-induced injurious mechanisms (**Table 1**) (2, 9, 20). While the relationship between CMV infection and allograft rejection has been well described, chemoprophylaxis against CMV was demonstrated to reduce rejection risk (21–24). Heterologous immunity describes the cross-reactivity of virus-specific memory T-cells to alloantigens and is a potential pathogen-driven mechanism of allosensitization and rejection (9, 25). Recurrent CMV infections and accumulation of cross-reactive T-cells through heterologous immunity may be a potential barrier to achieving graft tolerance (26–28).

**Table 1:** Possible mechanisms by which infection trigger rejection.

INFECTION LEADING TO REJECTION	
**Direct inflammation/tissue injury**	- CMV/BK - recruitment of proinflammatory mediators, Natural Killer (NK), CD8 & γδ T-cells (27, 35, 36). - Bacterial infection – Pyelonephritis (renal allograft rejection), pseudomonas *spp* (lung transplant rejection) immune infiltration and alloimmunity (3, 29)
**Modulation of surface proteins**	- Upregulation of adhesion molecules (VCAM/ICAM) increase immune cell infiltration and activation (9, 37–39). - MHC Class II receptors - allorecognition and T-cell activation (37, 40).
**Switching from regulatory to inflammatory T-cell phenotype**	- Reprogramming from regulatory T-cells to pro-inflammatory phenotype - CMV, non-commensal bacteria (9, 27, 41). - CMV associated accelerated T-cell ageing and switch to proinflammatory phenotype (36, 42, 43).
**Heterologous Immunity**	- Virus specific memory T-cells cross-react to self-antigens/HLA molecules (allosensitization) - CMV, EBV, VZV, Influenza A (9, 20, 44, 45). - Pathogen induced alloreactive memory T-cells shown to block ability to develop graft tolerance (26, 27).
***De novo* DSA formation**	- Potential association between viral infections (SARS-CoV-2, CMV/BK) and *denovo* DSA formation (30–33). - post-SARS-CoV-2 DSA, mainly restricted to those with high immunological risk and severe infections (31).
**Viral gene product modulation of immune responses**	- CMV infected monocytes - gene expression modification to proinflammatory phenotype and upregulation of chemokine expression (46). - Increase in interferon (IFN) signaling and cytotoxic T-cell function (47).
**Shock/Ischemia reperfusion injury**	- Damage associated molecular patterns trigger pro-inflammatory response (48–51). - Endothelial injury and inflammation (52–54).
**Immunosuppression reduction**	- Sepsis, CMV, BK nephropathy (high peak virus, calcineurin inhibitor (CNI) reduction >20%, MMF discontinuation) (4, 5). - BKN related IS reduction particularly assoc. with rejection (4).
**Virus related malignancies: Post-transplant lymphoproliferative disorder (PTLD) post-EBV**	- PTLD → Immunosuppression reduction to improve cytotoxic T-cell response against EBV induced B-cell clonal proliferation, associated with increased risk of rejection (55, 56).

CMV, cytomegalovirus; γδ, gamma delta T-cells; VCAM, vascular cell adhesion molecule; ICAM, intracellular cell adhesion molecule, MHC, major histocompatibility complex, SARS-CoV-2, severe acute respiratory syndrome coronavirus2, BK- human polyomavirus 1; EBV, Epstein Barr virus.

Direct graft inflammation and immune cell recruitment following infections with viral (CMV, BK) and bacterial pathogens (pyelonephritis) have also been linked to allograft rejection. However, the mechanisms by which rejection occurs remains poorly understood (2, 3, 9, 29). Lymphopenia is a common complication of CMV disease and/or its treatment. Immunosuppression modulation in response to lymphopenia, particularly mycophenolate dose reduction, may increase the risk of immune activation and rejection (10, 11).

Several studies demonstrate that immunosuppression modulation/interruption in the context of infection, particularly following BK viremia, increases the risk of rejection (4, 14). However, most studies on rejection risk following immunosuppression reduction or interruption are mixed (30–33). There may be an increased risk of *denovo* DSA formation and/or rejection in transplant recipients who experience more severe infection and require intensive and/or prolonged immunosuppression reduction and in those who are at high immunological risk (31, 34).

**Table 1** outlines the various mechanisms by which infection may elicit immunological changes that lead to rejection.

### 3.2 Rejection causing infection

Treatment of allograft rejection with lymphocyte-depleting therapies increases the risk of opportunistic infections that carry significant morbidity and mortality risk (14, 16). The infection risk is often prolonged with therapies such as anti-thymocyte globulin, where T-cell depletion can persist beyond one year. Immunosuppression-induced leukopenia/lymphopenia increases the risk of infections, particularly CMV (57–59). Furthermore, CMV and PJP chemoprophylaxis with agents such as valganciclovir and trimethoprim/sulfamethoxazole can also cause lymphopenia/neutropenia, and interruption of these agents to reverse the leukopenia can increase the susceptibility to these opportunistic infections (10, 11).

Transplant recipients with neutropenia experienced more bacterial infections and the degree of neutropenia correlated with infection risk (60). Several international guidelines recommend initiating CMV prophylaxis for at least three months following anti-rejection therapy with lymphocyte-depleting agents (61, 62). Intentional or accidental omission of chemoprophylaxis following potent anti-rejection immunosuppression may pave the way for viral reactivation and disease.

Chronic kidney disease, particularly late-stage kidney dysfunction, is associated with adaptive and innate immune system dysregulation and accelerated immune aging (63, 64). Shift towards exhausted and immunosenescent lymphocyte phenotype, CD4^+^ T-cell lymphopenia, reduced CD4^+^/CD8^+^ ratio, increased terminally differentiated T-cells, and chronic systemic inflammation are all described to be associated with uremia and progressive CKD (63, 64). Profound allograft dysfunction in the context of rejection, coupled with anti-rejection therapy, can compound the state of immunocompromise and hinder infection and vaccine-induced immune responses. Transplant recipients with lower eGFR (progressive dysfunction beyond <30mL/min/1.73m^2^) were less likely to achieve positive vaccine sero-response and require extra booster doses to achieve seroprotection (65).

### 3.3 Immunosuppression modulation during infection

**Figure 3:**
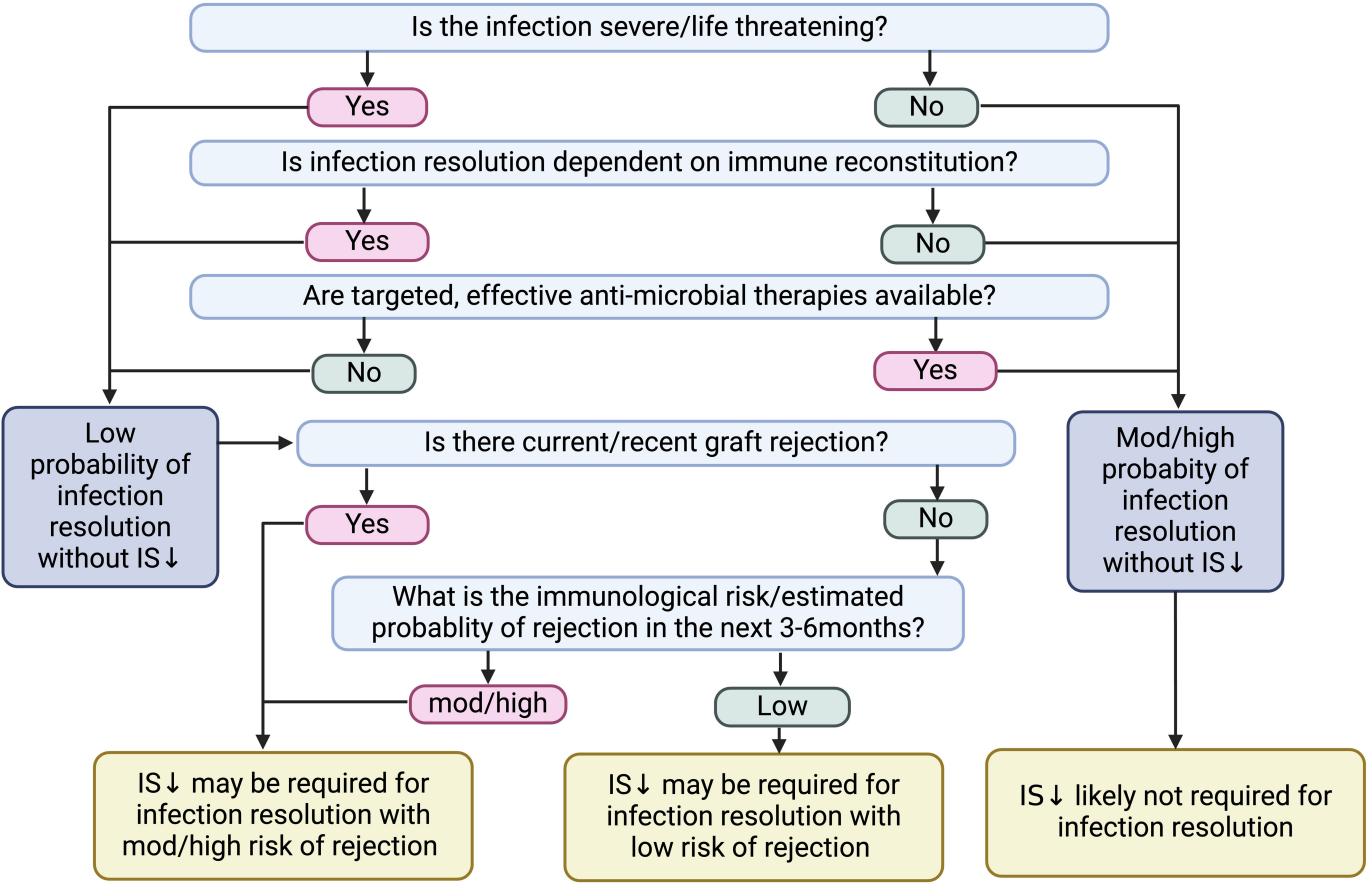
Factors to consider prior to immunosuppression reduction following infection. IS, immunosuppression.

The decision for immunosuppression reduction should consider infection severity, immunological risk of rejection, availability of targeted anti-microbial therapies, and the need for immune reconstitution for infection clearance (**Figure 3**) (14). Invasive fungal infections (e.g., *Cryptococcus* spp) and other severe opportunistic infections (e.g., *norcadia* spp) have considerable 1-year mortality rates and often require immune reconstitution for pathogen control.

While immunosuppression reduction following severe infection was associated with improved patient survival, most infections, particularly mild infections, did not require immunosuppression reduction for infection resolution (14). Additionally, there are no clear guidelines on the immunosuppression agents to reduce. Higher immunosuppression levels are required in the early post-transplantation period. Therefore, immunosuppression reduction in the early phase may be associated with a higher risk of rejection (4).

Most clinicians reduce anti-metabolites first, particularly in the setting of leukopenia, followed by calcineurin inhibitor (CNI) reduction. However, there are no evidence-based protocols on the best immunosuppression reduction strategy (66, 67). Individual immunosuppression agents have different impacts on the innate and adaptive immune systems and, as such, may have different risk profiles for infections with specific microbial organisms (14, 68). Understanding this may assist with clinical decision-making around immunosuppression modulation (14). Adopting a mammalian target of rapamycin (mTOR) inhibitor-based immunosuppression regimen is a common strategy, particularly following viremia. The mTOR inhibitors suppress viral replication and are associated with a reduced risk of viral respiratory infections, increased viral clearance following BK and CMV infections, and milder SARs-CoV-2 infections (69–72).

## 4 Current and emerging tools to measure infection and rejection risks

Given the lack of validated or standardized tests, clinicians rely on surrogate markers of immunity to determine the individual risk of rejection versus infection. Conventional organ-specific allograft dysfunction measurements are neither specific nor sensitive for rejection diagnosis and often lag behind intragraft immunological injury. Biopsy, the definite diagnostic tool for rejection, is invasive and imprecise and is subject to variations in sampling, processing, and pathologists’ reporting.

Immune markers provide insights into the innate and/or adaptive arms of the immune system (73–75). Some immune cells, such as NK cells, play a dual role in immune activation and graft tolerance (76). **Supplementary Table S1** provides an overview of the immunological markers of infection and rejection, including absolute lymphocyte counts and subsets (57, 58, 77–89), NK cell count and function (76, 90–96), immunoglobulin levels (37, 97–101), complements (102, 103), mannose-binding lectin levels (104), soluble CD30 (a transmembrane glycoprotein of the tumor necrosis factor family, cleaved from activated effector and memory T-cells) levels (74, 105–110), and CD4+ intracellular adenosine triphosphate (iATP) concentrations (75, 111–115). Lymphocyte number and subsets, immunoglobulin levels, CD4+ iATP concentrations, sCD30 levels, and cell-mediated immunity assays (IFN-γ release) reflect the adaptive immune response (73–75), whereas NK-cell number, complement components 3,4 (C3, C4) levels, and mannose-binding lectin (MBL) levels are markers related to innate immunity (73).

Viral load quantification describes the coordinated efforts of the innate and adaptive arms of the immune system (14). Current and historical drug levels are common non-immune-based surrogate markers of the immune state.

Several emerging biomarkers have been evaluated for use in the transplant population, however, their integration into routine clinical care requires standardization of assays and test thresholds, favorable test characteristics, clarification of clinical contexts of use, and optimization of costs and availability. A rapid turnaround time is also essential for the dynamic analysis of the immune state.

Despite the mounting evidence in favor of the use of certain biomarkers, to date widespread uptake has been restricted, primarily by the lack of external validation and standardization of commercial assays and diagnostic thresholds, excess costs and limited access. We will explore several emerging biomarkers of infection and rejection, emphasizing those with the greatest promise for clinical implementation.

### 4.1 Immune composite scoring systems

Several immune scoring systems have been evaluated to improve the infection-risk prediction. Dendle et al. (2018) describe a 4-point composite scoring system predicting severe infection risk in kidney transplant recipients (KTRs). Lower NK cell or CD4^+^ T-cell count, mycophenolate use, and lower eGFR positively correlated with the risk of severe infection (116). Crepin et al. (2016) examined an “immune risk profile (IRP)” defined by positive CMV status, CD4/CD8 ratio <1 and/or CD8 T-cell count >90^th^ percentile, which was predictive of opportunistic and spontaneous bacterial infections (117). The incidence of acute allograft rejection was lower in the IRP-positive group.

In kidney transplant recipients, the “simplicity score” calculated one month post-transplant was able to predict future risk of infections with good discrimination capacity (118). The score incorporates immune (C3 level, IgG level, CD4+ T-cell count, CD8+ T-cell count), and clinical (recipient age, glomerular filtration rate, recipient age, and infection within the first month) variables (118).

Sarmiento et al. (2014) described a composite “immunological score” using immunoglobulins (IgG, IgM, IgA), complements (C3, C4), and lymphocyte subsets (CD3+, CD4+, CD8+ T cells, NK cells, and B-cells) correlating with severe infections in a cohort of heart transplant recipients (119). IgG <600 mg/dL, C3 <80 mg/dL, C4 <18 mg/dL, NK count <30 cells/µL, and CD4 count <350 cells/µL were all associated with a significantly higher risk of infection, and these five parameters were used to derive the immunological score. Assigned points for each parameter totaled a maximum score of 16. An immune score ≥13 was associated with the highest risk of infection.

### 4.2 Quantification of viral loads

Several viruses, including CMV, EBV, and BK, are highly seroprevalent and remain latent until waning immune surveillance and clearance allows for viral replication and reactivation. Higher quantitative viral loads positively correlate with the intensity of immunosuppression (14, 120). Identifying and quantifying viral replication through nucleic acid amplification helps guide prophylactic, pre-emptive, and therapeutic treatment strategies. The emergence of viremia and/or rising viral loads suggests overimmunosuppression and waning viral surveillance (14). Viral infections lacking targeted therapies rely on robust immune responses. T-cell function is particularly important for viral suppression and clearance (14).

BK viral loads over 10,000 copies/mL increase the risk of BK nephropathy. Clinicians commonly use this threshold for immunosuppression reduction (121). Calcineurin level reduction >20%, mycophenolate discontinuation, and high peak BK viral loads correlate with an increased risk of allograft rejection (5). Higher EBV load is linked to PTLD development and the need for immunosuppression reduction (56).

Torque tenovirus (TTV) is a ubiquitous human virus of unclear pathogenic significance. It is unaffected by currently available antiviral therapies and, as such, is a valuable marker of immune competence (14, 122, 123). The kinetics of TTV can provide a measure of the integrated innate and adaptive immune responses. High TTV titres correlate with a reduced risk of allograft rejection and an increased risk of infection (124, 125). A prospective observational study by Doberer et al. (2020) demonstrated a 22% reduced risk of kidney allograft rejection and an 11% increased risk of infection with each log increase in TTV copies/mL (122). TTV counts were significantly higher in KTRs with bacterial, viral, and fungal infections, and elevated counts were detected up to 3 months before the infection (125, 126). A TTV viral load of >3.45 log DNA copies/ml within the first ten days post-transplantation positively predicted the risk of CMV reactivation (127). A TTV threshold level >1x10^6^ copies/ml could exclude rejection with a sensitivity of 94% (124).

KTRs with histopathological lesions of active rejection had lower TTV loads (128). Furthermore, the risk of developing histological features of chronic rejection was associated with the number of days with a TTV viral load <1x 10^6^ copies/ml between 3 to 12 months post-transplant, suggesting suboptimal immunosuppression (128).

### 4.3 Virus-specific cell mediated immunity

Measurement of virus-specific cell-mediated immunity (CMI) can provide insights into cellular immunocompetence and the ability to suppress viral replication. Understanding the strength of virus-specific responses can risk stratify transplant recipients and personalize chemoprophylaxis duration and monitoring (129–132). Immune assays commonly stimulate and measure T-cell functions, including activation, cytokine expression/production (IFN-γ), proliferation, and cytotoxicity (133).

Enzyme-linked immunospot, enzyme-linked immunosorbent assay, flow cytometry and intracellular cytokine staining (ICS), and MHC multimer staining (CMV CD8^+^ Immune Competence) are currently available techniques to assess viral-CMI (133). The flow cytometry ICS also allows for the co-staining of other cytokines and T-cell surface markers to characterize immune cell phenotypes.

CMV-CMI (QuantiFERON-CMV, ELISpot and ICS assays) correlated with functional T-cell responses and viral control (129–132). A positive QuantiFERON-CMV assay at the end of chemoprophylaxis in high-risk recipients (donor +ve/recipient -ve) yielded a positive predictive value of 90% for immune protection (132). Whereas indeterminant or negative results yielded the highest risk of CMV disease, likely correlating with blunted cellular responses (129–132). Quantiferon-CMV status was helpful for risk stratification of individuals with asymptomatic, low-level viremia following prophylaxis cessation (134). Those who were Quantiferon-CMV positive had a higher likelihood of spontaneous clearance, whilst those who were negative had a higher risk of CMV disease (134). Quantiferon-CMV guided pre-emptive therapy was more cost-effective than pre-emptive therapy alone (135). CMV-CMI responses were suppressed for up to 3 months post-ATG therapy. High dose prednisolone and elevated tacrolimus levels particularly impaired CMV-specific functional T-cell responses (129).

T-cell alloreactive CMI assays may be helpful in predicting immune activation and rejection risk, but studies to date are heterogeneous and quote different performance characteristics. Positive pre-transplant donor-reactive IFN-γ responses (ELISpot), particularly at higher levels, were associated with a greater risk of post-transplant rejection (136, 137). The meta-analysis by Montero et al. (2019) found that KTRs with positive pre-transplant donor-specific IFN-γ ELISPOT results had a 3.3-fold higher risk of acute rejection (138). A high number of donor-reactive memory T-cells, as measured by IFN-γ and interleukin-21 ELISPOT assays, was significantly associated with the risk of kidney allograft rejection (139).

CMI is an evolving additive tool for infection risk quantification, particularly for CMV infection. However, further research is needed to establish clear test thresholds relevant to different risk groups (recipient CMV positive vs. negative).

### 4.4 Medication/immunosuppression levels

Drug levels are often used to assess the overall immune state. However, the impact of the individual immunosuppressants on the overall immune state is difficult to quantify due to heterogeneity in individual drug pharmacokinetics and pharmacodynamics coupled with concomitant drug dosage adjustments. Therapeutic drug monitoring and target concentration intervention aims to optimize immunosuppression while minimizing toxicity. Calcineurin inhibitor and mTOR inhibitor levels are routinely measured for dose titration.

The proposed optimal target trough tacrolimus level for kidney allografts is 5-8ng/ml beyond the first few months post-transplant[128]. Lower levels are associated with rejection, and higher levels with infection and toxicity (140). CNI level variability relating to non-adherence or underdosing increased the risk of intragraft interstitial fibrosis/tubular atrophy, allograft rejection, and failure (121). Time in therapeutic range (TTR) >78% in the first year post-transplantation was associated with reduced rates of rejection and infection (141). Tacrolimus trough levels >10ng/ml were associated with an increased risk of BKVN, while lower levels were associated with rejection (142, 143). Every 1ng/mL increase in Tacrolimus trough levels beyond 5.35ng/mL at one month post-transplant was associated with an 11% higher rate of infections (144). Genetic polymorphisms related to drug clearance can play a critical role in rejection. Friebus-Kardash et al. (2022) demonstrated that CYP3A5 expressers achieved lower tacrolimus trough levels and were at greater risk of *de novo* DSA formation (145).

In contrast to CNI and mTOR level-based dose titration, mycophenolate dosing based on regular area under the concentration-time curve (MMF.AUC) measurements or Cmin (minimum concentration) levels have yet to be universally implemented. A fixed dosage mycophenolate regimen is standard practice in many transplant centers worldwide (146). Mycophenolate AUC measurements (targeting 30-60mg/L.h) are often used on a case-by-case basis, such as in the context of infection or malignancy, to guide dose adjustments. Mycophenolate AUC-based dose adjustment reduced infection rates in the 12 months post-kidney transplantation relative to a fixed-dose regimen (147). Individuals with high mycophenolate exposure (AUC 60-100mg/L.h) may safely and cautiously have their mycophenolate dose reduced (e.g. following leukopenia) while maintaining a level required for rejection prophylaxis. A mycophenolate AUC >50mg/L.h at three months post-transplant was associated with sustained BK viremia and BKVN in the subsequent two years (142). Conversely, mycophenolate AUC levels <30mg/L.h have been strongly correlated with rejection, particularly with other risk factors such as high immunological mismatch, delayed graft function, and low levels of concomitant immunosuppressants (148, 149). Despite this, most studies suggest an increased risk of rejection below a threshold of 40mg/L.h, particularly beyond six months post-transplantation (146). Reduction or discontinuation of mycophenolate, irrespective of tacrolimus levels, was associated with adverse graft outcomes, including rejection (146, 150). Hence, the impact of individual drug levels on the overall immune state warrants consideration.

### 4.5 Donor specific antibody detection (HLA and non-HLA)

Anti-HLA donor-specific antibodies may be pre-formed or *de novo* (develop post-transplantation). The detection of donor-specific antibodies (against HLA antigens) has long been the hallmark of the diagnosis of antibody-mediated rejection (AMR) according to the BANFF criteria (151). Recipients with pre-formed antibodies at the time of transplant are at a higher risk of AMR. Within five years post-transplant, 15-25% of transplant recipients develop *de novo* DSA, with an incidence of 2% per year in immunosuppression adherent transplant recipients (152, 153). The DSA titer, as measured by the mean fluorescence intensity (MFI), correlated strongly with the risk of antibody-mediated rejection and graft failure (154).

This correlation is especially true of complement activating (C1q-positive) and anti-HLA class II DSA (155, 156). There is emerging evidence on the significance of several non-HLA antibodies, including those against major histocompatibility complex class 1-related chain A (MICA), type I angiotensin II receptor, endothelin A receptor, and collagen, in the development of AMR (157).

Under-immunosuppression, owing to medication dosing, genetic variability in drug metabolism, or non-adherence strongly favors DSA formation (152, 158, 159). A high degree of HLA mismatches (especially DQ) and events that cause graft inflammation and increased immunogenicity, including ischemic injury, infections, and cellular rejection, can also trigger *de novo* DSA formation (154, 160–162).

During the first-year post-transplantation, *de novo* DSA formation was more common in transplant recipients with subtherapeutic tacrolimus levels (<5ng/ml), particularly in the preceding 6 months (163, 164). Additionally, achieving less than 60% of the time within the therapeutic range (5-10ng/mL) in the first year post-transplant was associated with an increased risk of *de novo* DSA formation, rejection at 12 months, and graft loss by five years (143).

With the advent of molecular diagnostics, DSA-negative AMR was revealed to be a more common AMR phenotype than previously recognized (165). Several minimally invasive biomarkers may detect graft injury and assist in diagnosing AMR without clear indicators, such as DSA.

### 4.6 Donor-derived cell-free deoxyribonucleic acid

Donor-derived cell-free DNA (dd-cfDNA) is one of the most promising and extensively studied biomarkers. Also termed the “liquid biopsy,” dd-cfDNA is a useful screening tool to identify individuals at risk of rejection, who would benefit from a definitive histological diagnosis. A threshold of 1% dd-cfDNA distinguished allograft injury and rejection (166–169). Higher levels of dd-cfDNA correlated with a greater degree of histological injury (170).

Following cell apoptosis and necrosis, a small proportion of DNA, termed cell-free DNA, enters the circulation. Graft tissue necrosis was associated with larger fragment cfDNA (10,000 base pairs), while apoptosis with smaller fragments (60-500bp) (168). In transplant recipients, a small fraction of dd-cfDNA may enter the recipient’s circulation following graft injury (171). Urinary dd-cfDNA can arise from glomerular filtration of circulating dd-cfDNA or donor DNA released from the donor urinary tract (169, 172). Dd-cfDNA can be quantified as relative (proportion (%) of total cf-DNA) or absolute quantitative dd-cfDNA (cp/ml). The diagnostic performance of dd-cfDNA varies depending on the rejection phenotypes, clinical context, immunological risk, and assay type.

Several local and systemic causes affecting graft integrity can elevate circulating dd-cfDNA levels, including infections, calcineurin inhibitor-induced nephropathy, disease recurrence, and ischemia/acute tubular necrosis (167). As such, all potential causes of allograft injury must be considered when assessing dd-cfDNA elevation.

#### 4.6.1 Donor-derived cell-free DNA and infection

KTRs with infections that compromised graft integrity, including BK nephropathy and urinary tract infections, had elevated plasma dd-cfDNA levels (173). A dd-cfDNA rise of >1% was noted within seven days of a respiratory viral infection (RVI) in lung transplant recipients (174). In lung transplant recipients with RVI, a greater plasma %dd-cfDNA rise correlated with poorer lung function recovery post-RVI (174). In a prospective cohort study of 44 heart transplant recipients, those who tested positive for CMV infection had significantly higher plasma dd-cfDNA levels as compared to those who were CMV-negative (175). Dd-cfDNA elevations have also been described in case studies of heart transplant recipients with myocardial injury following COVID-19 infection (176).

#### 4.6.2 Donor-derived cell-free DNA and rejection

##### 4.6.2.1 Liver transplant

In a multicenter cohort study of 219 liver transplant recipients, serial plasma dd-cfDNA elevations accurately predicted rejection in recipients with normal liver function tests (177). Additionally, dd-cfDNA levels decreased following successful rejection treatment. Day 7 dd-cfDNA level >10.2% accurately predicted the risk of early rejection within the first three months post-transplant (sensitivity-93%, specificity-94%, positive predictive value (PPV)- 88%, negative predictive value (NPV)-97%) (178).

##### 4.6.2.2 Lung transplants

The systematic review by Li et al.(2023) demonstrated that elevated plasma dd-cfDNA in lung transplant recipients distinguished rejection versus no-rejection with high pooled sensitivity and specificity of 87% (95% CI: 80-92%) and 82% (95% CI: 76-86%) respectively (179). Dd-cfDNA was elevated in lung transplant recipients with subclinical rejection (AMR & TCMR) and infection (180, 181). A multicenter retrospective cohort study by Keller et al. (2022) assessed the performance characteristics of plasma dd-cfDNA (≥ 1%) in detecting acute lung allograft dysfunction, a composite marker of infection and rejection, in 175 asymptomatic lung transplant recipients. Sensitivity, specificity, PPV, and NPV of dd-cfDNA ≥1% were 74%, 88%, 43%, and 97%, respectively (181). Given the very high NPV, a normal dd-cfDNA may be helpful in excluding underlying rejection or infection in stable lung transplant recipients.

##### 4.6.2.3 Heart transplant

In a prospective cohort study of 223 heart transplant recipients, dd-cfDNA of ≥0.15% accurately predicted rejection with sensitivity, specificity, PPV, and NPV of 79%, 77%, 25%, and 97%, respectively (182). The specificity for rejection was slightly higher (82%) with a ≥0.2% threshold, however the PPV remained poor (30%). In a multicenter-prospective cohort study of 740 heart transplant recipients, those who had any-cause rejection had higher plasma %dd-cfDNA than those who did not (median: 0.17% vs. 0.07%) (183). Despite a poor sensitivity (44%), a threshold of >0.2% demonstrated a very high NPV (97%) for allograft rejection.

##### 4.6.2.4 Kidney transplants

The addition of dd-cfDNA to standard diagnostic algorithms greatly enhanced their discriminatory power for AMR, TCMR, and mixed rejection phenotypes (170). However, dd-cfDNA can better predict AMR than TCMR. Additionally, the magnitude of dd-cfDNA correlated with the degree of graft injury and rejection severity (166–168, 170, 173).

Owing to significant heterogeneity amongst studies, a wide range of performance metrics for dd-cfDNA in diagnosing kidney allograft rejection has been reported. A systematic review and meta-analysis by Xing et al. (2024) included nine studies assessing the accuracy of plasma dd-cfDNA in diagnosing any rejection and 12 studies specific to AMR (184). The diagnostic accuracy of dd-cfDNA was much greater for AMR as compared to any-rejection phenotypes. The pooled sensitivity was only 59% (95% CI, 48–69%) for any-rejection diagnosis; however, the specificity and the area under the receiver operating characteristics curve (AUROC) were more favorable at 83% (95% CI,76–0.88%) and 80% (95% CI,76–83%) respectively. Comparatively, the pooled sensitivity, specificity, and AUROC for AMR diagnosis were 81% (95% CI, 72–88%), 80% (95% CI, 73–86%), and 87 (95% CI, 84–90%), respectively. “The European Society of Organ Transplantation’s (ESOT) Consensus Statement on Non-invasive Diagnosis of Kidney Allograft Rejection” favored the use of dd-cfDNA in transplant recipients with allograft dysfunction to exclude rejection, particularly AMR (185). The magnitude of plasma dd-cfDNA coupled with histological features may help re-classify rejection severity and predict clinical outcomes such as eGFR decline and risk of *de novo* DSA formation (186, 187). The prospective, multicenter Trifecta study by Halloran et al. (2023) assessed the relationship between plasma dd-cfDNA, DSA, and molecular signatures in 280 kidney transplant biopsies (188). DSA-negative AMR was more prevalent than previously described, as 56% of the molecular and 51% of the histological AMR diagnoses were DSA-negative. DSA-negative and positive AMR had similar degrees of dd-cfDNA elevation, making dd-cfDNA a useful predictive tool for identifying AMR in the absence of DSA and prompting a confirmatory allograft biopsy (188). Screening with dd-cfDNA may not be reliable for detecting subclinical or low-grade T-cell mediated rejection (type 1 A) (166, 185). Compared to AMR, the fractional plasma dd-cfDNA threshold of 1% was less sensitive for TCMR, which required a higher diagnostic threshold (189). However, combining dd-cfDNA with other biomarkers such as molecular markers may improve the overall test accuracy for predicting TCMR. The dynamics of dd-cfDNA in the early postoperative period is unclear, and several confounding factors that cause graft injury can elevate the levels during this time. As such, dd-cfDNA is not particularly useful for rejection-infection diagnosis in the immediate peri-operative period. Shen et al. (2019) assessed the dd-cfDNA fluctuations in the first two weeks post- kidney transplant (190). Deceased donor grafts had higher plasma %dd-cfDNA immediately post-transplant as compared to living donor grafts (45% vs. 10%), and those with delayed graft function had a slower decline in dd-cfDNA. A sudden rebound of dd-cfDNA levels may point to rejection as a possible cause.

With respect to diagnosing rejection in different SOT populations, most studies report higher sensitivity than specificity and a much higher negative predictive value (>90%) than a positive predictive value (191). As such, dd-cfDNA is particularly useful for ruling out allograft rejection (168, 169). Additionally, given the short half-life of circulating cfDNA (30-120 minutes), the return of dd-cfDNA levels to baseline can indicate successful treatment of allograft injury (rejection or infection), allowing for real-time assessment of allograft injury and recovery (192, 193).

Studies to date have not examined the use of dd-cfDNA in place of biopsies to diagnose rejection or assess longer-term clinical outcomes following rejection. Additionally, dd-cfDNA cannot discriminate between different rejection phenotypes. Dd-cfDNA can be useful for identifying transplant recipients with allograft injury who require a histological diagnosis whilst considering alternative causes, including local and systemic infections. Integration of dd-cfDNA into routine clinical care is gaining momentum, with commercial assays currently available in several countries in Europe and the United States. In the United States, dd-cfDNA has been Medicare reimbursable since 2017 (194). Despite its’ commercial availability, concerns regarding its poor specificity, assay validation, and significant associated costs have limited its uptake elsewhere including in Europe and Australasia. While the cost and availability currently prohibit widespread adoption of dd-cfDNA, there may be long-term cost savings if its integration as a surveillance tool leads to earlier histological rejection diagnosis and therapeutic intervention before chronic and permanent allograft damage ensues (168).

### 4.7 Urinary chemokines

Urinary chemokines are gaining significant ground as a valuable biomarker in assessing kidney allograft health. CXC-motif chemokine ligands 9 and 10 (CXCL9 and CXCL10) are interferon-γ induced chemokines that promote leukocyte migration and infiltration during allograft rejection (195, 196).

#### 4.7.1 Urinary chemokines and infection

Urinary chemokines are not specific for rejection, but rather, renal inflammation, and may also be elevated in KTRs with local infections, including BK nephropathy and urinary tract infections (197, 198). In a longitudinal study of 60 KTRs with BK viremia, urinary CXCL10/cr levels were identified as a prognostic marker of graft dysfunction and predicted the degree of infection related renal inflammation (199). A threshold value of 12.86ng/mmol was associated with a greater degree of inflammatory burden and eGFR decline. Urinary CXCL10 levels were not elevated with CMV viremia alone, highlighting that renal-specific inflammation was required to trigger urinary chemokine elevation (200).

Given the association between urinary chemokines and renal inflammation, all potential causes, including infection, must be considered and excluded before attributing the rise to rejection.

#### 4.7.2 Urinary chemokines and kidney allograft rejection

The reported sensitivity and specificity of both urinary chemokines in diagnosing rejection vary widely owing to different test thresholds and variations in the prevalence of rejection phenotypes in study populations. Reported CXCL9 sensitivity and specificity for detecting kidney allograft rejection ranged between 58%-86% and 64%-80%, respectively, and CXCL10 sensitivity and specificity from 59-80% and 76%-90%, respectively (195–197, 200). The high negative predictive value of CXCL9 and CXCL10 make them useful for ruling out acute kidney allograft rejection at low levels (195). Combination of both CXCL9 and CXCL10 did not provide superior discriminatory power compared to measuring individual levels (197).

Urinary CXCL9 and CXCL10 could accurately discriminate between allograft dysfunction due to rejection and non-rejection causes but were not able to distinguish between AMR and TCMR (195, 196). They may serve as an early indicator of allograft dysfunction with elevated CXCL9 levels detected up to 30 days before clinical changes and biopsy-confirmed acute rejection (195). Whereas lower urinary chemokine levels at 1- and 3-months post-transplant were associated with immunological quiescence and a lower risk of allograft rejection (201). Post-rejection monitoring with urinary chemokines identified individuals at risk of rejection recurrence. Low CXCL9 levels six months following a rejection episode correlated with a reduced risk of rejection in the subsequent 18 months (NPV 99.3%). Conversely, a rising CXCL10, a potential sign of persistent inflammation, was linked to eGFR decline (202). Successful treatment of allograft rejection was associated with reduced CXCL10 levels (197). CXCL10 elevations may reflect renal compartment-specific histological injury, as elevations accompanied tubulointerstitial inflammation and peritubular capillaritis but not isolated glomerulitis or vascular inflammation (200).

#### 4.7.3 Chemokines in other solid organ transplants

Plasma and tissue chemokines may serve as potential indicators of graft injury in other solid organ transplants, however their value in allograft rejection diagnosis has not been established. Plasma and bronchoalveolar lavage CXCL9 and CXCL10 levels were shown to positively correlate with chronic lung allograft damage and acute rejection in a prospective multicenter study of 184 lung transplant recipients (203). Liver transplants with early allograft dysfunction had elevated plasma levels of T-lymphocyte-associated chemokines and cytokines including CXCL9/CXCL10, in the early postoperative period (204). Inhibition of plasma CXCL9/CXCL10 levels delayed cardiac allograft rejection in murine models (205).

The ESOT consensus statement on non-invasive diagnostic tests recommended the use of urinary CXCL9 and CXCL10 to exclude or consider kidney allograft rejection in transplant recipients with acute allograft dysfunction (185). Whilst urinary chemokines show great promise as a serial surveillance tool, given that they are easy to access and non-invasive, they are currently not yet recommended for use in subclinical rejection (185). The randomized controlled trial by Hirt-Minkowski et al. assessed the utility of a serial urinary CXCL10 monitoring-based care in reducing poor graft outcomes at 1-year post-kidney transplant (206). CXCL10 monitoring did not reduce the primary endpoints of allograft rejection, death-censored graft loss, denovo DSA formation, or eGFR decline to <25 ml/min. Several prospective studies are currently underway that may shed light on the utility of serial CXCL9 and CXCL10 monitoring in predicting clinical and subclinical rejection.

### 4.8 Molecular markers/transcriptomics

Molecular diagnostics is a rapidly evolving field that could change the landscape of precision medicine by providing mechanistic insights into immune cell phenotypes and molecular pathways involved in allograft disease states. Gene expression changes in blood, peripheral blood mononuclear cells (PBMC), urine and tissue have been described in relation to various microbial infections and rejection phenotypes.

#### 4.8.1 Molecular markers of infection

Measurement of characteristic gene signatures may help clinicians tailor chemoprophylaxis and guide testing for viral reactivation. Ahn et al (2021) describe changes in whole-blood gene expression in CMV-positive KTRs across multiple time points within the first year post-transplantation (47). Peak gene expression differences occurred between baseline and 1-week timepoints involving the innate and adaptive arms of the immune system (e.g., interferon signaling and cytotoxic T-cells). While many pathways normalized post-infection, several genes remained differentially expressed at one year, suggesting long-term adaptations to the immune system (47).

Given similarities in histological features, molecular markers may prove useful in overcoming the diagnostic dilemma of rejection versus BK Nephropathy of the kidney allograft. Adam et al. (2020) described a five-gene set (Agnoprotein, LTAg, VP1, VP2, VP3*)* that reliably distinguished BK virus nephropathy from TCMR in biopsy specimens (207). Gene expression biomarkers may help differentiate between rejection and viral or bacterial infections in lung transplant recipients with acute respiratory symptoms (208, 209).

#### 4.8.2 Molecular markers of rejection

Molecular markers of subclinical rejection would help facilitate early diagnosis and treatment, ultimately reducing the risk of chronic damage and allograft loss.

The Molecular Microscope Diagnostic System (MMDx) project collates genome-wide microarray data to describe different molecular phenotypes (e.g. AMR, TCMR, parenchymal injury, irreversible atrophy-fibrosis) associated with allografts. Several molecular AMR phenotypes have been identified, including subclinical, DSA-negative, and C4d-negative subtypes (210). Molecular disease classifiers were able to discriminate between the rejection phenotypes (AMR, TCMR, mixed) in cases where histology was ambiguous (211). Halloran et al. (2024) identified gene transcripts that were TCMR and AMR selective and those shared by both rejection phenotypes. IFN-γ inducible (CXCL11, WARS, IDO1, and GBP4), effector T cells, and NK cell-related transcripts (KLRD1 and CCL4) were common to both AMR and TCMR phenotypes (210). The top TCMR-selective transcripts were predominantly associated with activated effector T-cells (IFNG, LAG3, SIRPG), macrophages, and dendritic cells (ADAMDEC1, CXCL13, CD86, and SLAMF8) (210). Some IFN-γ-inducible transcripts (ANKRD22 and AIM2) were highly selective for TCMR. NK cell (CD160, GNLY, KLRD1, SHD2D1B, CX3CR1) transcripts strongly correlated with AMR, suggesting a prominent role of NK-induced cell-mediated cytotoxicity in its pathogenesis (210). Endothelial cell (CCL4, DARC/ACKR1, CDH5, CDH13, COL13A1, etc.) and IFN-γ inducible gene transcripts (CXCL11, CXCL10, PLA1A) are also commonly described to be associated with AMR (165, 212, 213). AMR molecular classifiers closely correlated with microcirculation lesions, histological damage, and DSA (210).

The BANFF Molecular Diagnostics Working Group (MDWG) compiled a validated 770-gene BANFF-Human Organ Transplant (B-HOT) panel, which incorporated genes involved in immune responses, rejection, tolerance, and viral infections (214). The panel uses the NanoString nCounter platform, which allows relatively rapid quantification of transcripts from fresh or formalin-fixed paraffin-embedded samples.

Blood gene expression profiling (GEP) (commercial assay-AlloMap^®^) has been extensively studied, primarily in heart transplant recipients, to reduce the need for frequent endomyocardial biopsies. The 11 rejection-associated gene set was discovered and validated in the CARGO study, a prospective observational study of heart transplant recipients (215). A score beyond a determined threshold was associated with a higher likelihood of acute cellular rejection in cardiac allografts (215). The IMAGE and EIMAGE trials revealed non-inferiority of GEP compared to a biopsy-driven protocol for detecting rejection (216, 217). The Outcome AlloMap registry, a multicenter prospective study of 1504 heart transplant recipients, showed that GEP surveillance was associated with improved survival, reduced rates of allograft dysfunction, and acute rejection (218). Low 2-6 months and >6-month post-transplant GEP scores had NPVs for rejection of 98.4% and 98.5% respectively. Kidney-specific GEP (5-gene classifier: DCAF12, MARCH8, FLT3, IL1R2, and PDCD1) discriminated between immune quiescence and rejection (219).

The Kidney Solid Organ Response test (kSORT) assay is a peripheral blood 17 gene-set panel associated with rejection (220). Whilst the original study, the Acute Rejection in Renal Transplantation (AART) study of 436 KTRs, reported a high PPV (81.3%–95.5%) and NPV (91.6%–98.0%) for detecting rejection, this finding was not validated in a subsequent retrospective multicenter study of 1763 KTRs (220, 221). Several studies since have yielded conflicting results.

TruGraf^®^v1 is another commercially available blood 200-probe micro-array gene-expression signature assay that is useful in stable allografts to identify subclinical rejection (222). A positive test was associated with poorer 24-month allograft outcomes and the development of DSA (222). The application of an 11-gene set signature, termed the “common rejection module” on urine and tissue (kidney allograft biopsies) was shown to discriminate between stable and rejection biopsies (223–225).

Given the comparative ease of collection relative to biopsies, blood and urine gene expression profiling may be a helpful adjunct in diagnostic algorithms. Despite the promising studies, ESOT guidelines currently do not recommend the clinical use of gene expression signatures to diagnose allograft rejection in those with acute allograft dysfunction (185). The issue lies in the absence of clearly defined, validated, and reproducible gene set signatures that can distinguish between rejection and no rejection, and differentiate between rejection phenotypes. Furthermore, the gene sets need to be specific to the biological specimen type. Identifying specific gene sets in more extensive, prospective studies, improving costs and availability, and standardizing testing may push molecular diagnostics to the forefront of rejection-infection diagnosis. The MMDx project and the global collaboration of gene transcript research using the Nanostring B-HOT panel may help with this endeavor (214). Molecular profiling may allow for the re-classification of rejection phenotypes and severity, particularly where histology is ambiguous. Additionally, gene signatures can provide insights into intragraft changes associated with rejection therapies.

#### 4.8.3 MicroRNAs

MicroRNAs (miRNA) are short (22 nucleotides), non-coding RNA segments that regulate gene expression and play an important role in modulating homeostatic and disease processes, including allograft rejection (226, 227). Up to 60% of transcribed genes of the human genome are targeted by miRNA, and different cell types express specific subsets of miRNA (227). MiRNAs may also provide organ and disease-specific signatures relevant to allograft rejection and infection. MiRNAs may be up- or down-regulated and detected in the systemic circulation (serum or PBMC) or urine using real-time PCR, microarray and next-generation sequencing (228). They are annotated by a “miR” prefix and a unique number identifier (229).

##### 4.8.3.1 miRNA in infection

Whilst miRNAs have predominantly been assessed with respect to rejection, miRNA profiling may also assist with infection prediction and diagnosis. Viral miRNAs regulate viral genes relating to replication, immune evasion and viral persistence and can be detected in biological samples.

Two BK-virus miRNAs, bkv-miR-3p and bkv-miR-5p, regulate viral replication. Serum levels of these miRNAs were higher in KTRs with BK Nephropathy (230). Demey et al. (2021) demonstrated that in KTRs with BK viremia, urinary bkv-miR-B1-3p and bkv-miR-B1-5p levels reduced in concert with reductions in serum viral loads (231). Additionally, increased urine bkv-miR-B1-5p levels suggested active viral replication (232). BK-virus encoded miRNA bkv-miR-5p and bkv-miR-3p downregulate viral large T-antigen (LTag) expression, reducing viral recognition and allowing immune evasion and persistence (230). Downregulation of ULBP3 by BK-virus encoded miRNA dampened NK-cell mediated killing of viral-infected cells (233).

Several miRNAs are described to be associated with CMV viremia and infection. Afshari et al. (2022) found that KTRs with CMV viremia had significantly higher plasma expression of the CMV-encoded miRNAs, miR-UL112-3p/5p, miR-UL22A-3p/5p, miR-US25-1-5p, miR-US25-2-3p/5p, miR-UL36-3p/5p and miR-UL70-3p, relative to those with latent CMV (234). Reduced expression of miR-125a-5p in CMV seropositive KTRs with positive CMV-specific cell-mediated immunity predicted those who were at higher risk of CMV infection despite the positive CMI result (235).

Other microbial pathogens also induce characteristic miRNA signatures. In bronchoalveolar lavage (BAL) samples of lung transplant recipients, increased levels of miR-23b-3p expression were associated with pneumonia (236). Dysregulation of five miRNAs (miR-145-5p, miR-424-5p, miR-99b-5p, miR-4488, and miR-4454/miR-7975) in BAL specimen was specific for invasive aspergillosis in lung transplant recipients (237). A 25-set intragraft miRNA signature differentiated acute pyelonephritis from allograft rejection in KTRs (238).

##### 4.8.3.2 miRNA in rejection

Studies have associated miRNA expression with immune cell pathways implicated in rejection, notably T-cell activation and regulation. FOXO1 is a key regulator of several cellular and immune cell processes and plays a critical role in the development of FOXP3 regulatory T-cells (226). Increased miR-182-5p expression following IL-2 and STAT5 activation, has been shown to suppress FOXO1 and reduce regulatory T-cell production (226). Elevated miR-182-5p levels were also noted in mice with rejecting cardiac allografts (239). Another miRNA, miR-146a was significantly upregulated in activated T-cells, particularly memory T-cells (226).

##### 4.8.3.3 Liver transplants

Several hepatocyte-derived miRNAs have been investigated for their potential to diagnose clinical and subclinical liver transplant rejection. In liver transplant recipients (LTRs), plasma miRNAs, miR-122, miR-148a, miR-194, were linked to liver injury and acute rejection (240). Plasma miR-483-3p and miR-885-5p levels could predict rejection in those undergoing immunosuppression withdrawal (241). Pre-transplant plasma miR-155-5p and post-transplant miR-155-5p and miR-181a-5p expression correlated with the risk of developing rejection in LTRs (242). These miRNAs may be helpful in stratifying immunological risk pre-transplant and rejection risk post-transplant (242).

##### 4.8.3.4 Heart transplant

Myocardium-specific miRNAs have also been linked to cardiac allograft rejection. The multicenter prospective cohort study, the Genomic Research Alliance for Transplantation (GRAfT), identified 12 plasma miRNAs that predicted acute cellular rejection (ACR) (AUROC-0.92, 95% CI:0.86-0.98) and 17 that predicted antibody-mediated rejection (AUROC-0.82, 95% CI:0.74-0.90) (243). Other circulating miRNAs including miR-486-5p and miR-181a-5p have also been shown to predict ACR (244, 245). Circulating miR-10a, miR-92s, and miR-155 had previously been linked to cellular rejection, however the prospective multicenter study by Coutance et al. (2023) showed no associations between these three miRNAs and allograft rejection (246).

##### 4.8.3.5 Lung transplant

Epithelial-to-mesenchymal transition (EMT) is important in the pathophysiology of chronic lung allograft dysfunction (CLAD). Increased expression of circulating miR-21, a regulator of EMT, was seen in lung transplants with CLAD (247). TGF-β, is a potent inducer of EMT and a critical mediator of fibrosis. TGF-β associated miRNAs, miR-369-5p and miR-144, were dysregulated in lung transplant recipients with DSA and bronchiolitis obliterans syndrome (BOS) (248, 249). The miRNA signature of mononuclear cells (miR-369-5p, miR-144, miR-134, miR-10a, miR-195 miR-142-5p, miR-133b, and miR-155) was able to predict the development of DSA and BOS (248, 249). In bronchoalveolar lavage samples, low miR-148b-5p and high miR-744-3p expression distinguished rejection from no-rejection and was significantly associated with shorter time to the acute rejection episode (236).

##### 4.8.3.6 Kidney transplant

Urinary and blood miR-155-5p expression has been evaluated as a diagnostic and prognostic marker of allograft rejection in KTRs. Urinary miR-155-5p levels correlated with eGFR levels and normalized after successful treatment and resolution of allograft injury (250). Elevation in plasma miR-155, miR-223, and miR-21 expression was associated with AMR (251). MiR-223 was also raised in TCMR, alongside miR-142-3p, miR-10a, and miR-100a, whilst miR-99a declined (252). Changes in miRNA expression could precede clinical and histological features of TCMR, with elevation in miR-155-5p and miR-142 expression and reduction in miR-210-3p expression noted before and during TCMR (250).

Whilst several studies have linked specific miRNAs with allograft injury and damage, consistent associations are yet to be validated. Available studies vary substantially with respect to study populations, sample type and size, detection assays, and thresholds used and demonstrate conflicting results. When tissue-specific, unique miRNA signatures are established, miRNA profiling may be a valuable additive tool for allograft surveillance and rejection prediction.

#### 4.8.4 Exomes

Exomes are nanometer-sized (50-200nm) extracellular vesicles that carry proteins, lipids, metabolites, and/or nucleic acids (mRNA or miRNA) and play a key role in inter-cell communication (253). They are present in almost all biofluids, and their composition and function are specific to the originating cell and are modulated by physiological conditions and stressors. The role of exomes in SOT has gained considerable interest. Exosomes that transport donor and self-antigens may play a key role in allorecognition and rejection (254). Urinary and plasma exome contents, particularly proteomic and nucleic acid profiles, may provide valuable insights into the underlying pathological processes such as rejection.

##### 4.8.4.1 Kidney transplant

In a prospective study of 175 KTRs, a urinary mRNA exome signature (CXCL11, CD74, IL32, STAT1, CXCL14, SERPINA1, B2M, C3, PYCARD, BMP7, TBP, NAMPT, IFNGR1, IRAK2, and IL18BP) distinguished any-cause rejection with no rejection (255).

The sensitivity, specificity, AUROC, and NPV were 85% (95% CI, 74 -92%), 94% (95%CI 88 – 97%), 93% (95% CI 87 – 98%) and 93% (95% CI 88 – 96%) respectively. Additionally, another specific gene signature (CD74, C3, CXCL11, CD44, and IFNAR2) distinguished TCMR and AMR phenotypes, with a AUROC of 0.87 (95% CI, 0.76 to 0.97). Eleven urinary exome proteins were enriched in KTRs with rejection (256). Tower et al. revealed an increase in the plasma concentrations of C4d^+^/CD144^+^ microvesicles, an endothelial marker, in KTRs with AMR (257). Additionally, C4d^+^/CD144^+^ microvesicle levels decreased following anti-rejection therapy.

##### 4.8.4.2 Heart transplant

A serum proteomic profile of 15 exomal proteins distinguish cardiac allograft rejection with no-rejection. These related to complement activation (C1QA, C1R), coagulation (FIBA, FIBB, FIBG, FINC, F13A, and TSP1), IgG subfraction (KV302, HV304, HV315) and APOL1 (258). In a prospective study of 10 heart transplant recipients, serum exosomal miRNAs, miR-142-3p, miR-92a-3p, miR-339-3p, and miR-21-5p were enriched in individuals with allograft rejection (259).

##### 4.8.4.3 Lung transplant

Circulating exomes containing self-antigens may identify lung transplant recipients at risk of allograft rejection and CALD (260). In an observational study of 30 lung transplant recipients, donor-HLA and self-antigens were detected in bronchioalveolar lavage and serum exosomes of recipients with rejection and BOS but not in stable patients. Exomes containing Col-V and immunomodulatory miRNA were also isolated in lung transplant recipients with rejection (261).

Further large-scale research is required to validate and reproduce relevant organ specific exomes that may serve as biomarkers of organ health.

### 4.9 Integrating biomarkers

Integrating several markers, including molecular, immune, serological, and clinical parameters, may strengthen the overall diagnostic and predictive accuracy for rejection compared to a single method used in isolation. The combination of dd-cfDNA with gene expression profiling increased the predictive power for detecting allograft rejection (262). Additionally, in the study by Park et al. (2021) GEP detected more TCMR whilst dd-cfDNA detected more AMR (262). In a heart transplant cohort, compared to GEP alone for rejection surveillance, GEP with dd-cfDNA produced similar one year rejection-free survival but did so with significantly fewer biopsies (263). Integrating a pre-transplant functional immune assay (donor-alloreactive IFN-γ release assay/ELIspot) with the six-month post-transplant blood gene-expression kSORT assay was shown to predict subclinical rejection (264).

An integrated model combining urinary chemokines, CXCL9, and CXCL10 with six other clinical parameters (recipient age, gender, eGFR, DSA, signs of urinary tract infection, blood BK viral load) showed moderate diagnostic accuracy (AUROC, 0.85) for the detection of kidney allograft rejection (198). Similarly, an integrated model combining CXCL9, CXCL10 with clinical markers (eGFR, DSA, and BK viremia) also showed similar diagnostic performance for detecting acute rejection (AUROC-0.81) (265). Moreover, using the latter composite score, when the predicted rejection risk was <10%, 59 out of 100 protocol biopsies were avoided (265).

The combination of chemokines and miRNA profiling has also been assessed. Millan et al. (2023) described an integrated plasmatic model including three miRNAs (miR-155, miR-181a-5p, miR-122-5p) and CXCL10, which predicted TCMR in liver transplant recipients with good diagnostic performance, AUROC 0.98 and high PPV-97.7% and NPV-97.1% (266). In another study, an integrated model of miR-155-5p, miR-615-3p, and CXCL-9 had strong diagnostic accuracy for predicting kidney transplant allograft rejection (AUROC- 0.92) (267).

## 5 Simultaneous infection and rejection risk assessments and clinical application of biomarkers

Several tools support the assessment of the transplant recipients’ net immune state and help refine infection-rejection risk stratification. Considering the complex interplay between these two complications, their risks should be assessed concurrently (**Table 2**). Furthermore, the dynamic nature of the immune state necessitates regular assessments tailored to the clinical contexts. Several biomarkers, including dd-cfDNA, urinary chemokines, and miRNAs, lack the specificity for the prediction and diagnosis of rejection and may be deranged in the context of other confounding contributors that cause graft inflammation, including local and systemic infections (BK, CMV, UTIs, pneumonia). Consequently, the distinction between these entities requires clinical evaluation and assessment to identify the most probable clinical scenario. Additionally, both rejection and infection may co-exist. **Table 2** summarizes the currently available and emerging immune and non-immune tools for simultaneous infection and rejection risk assessments.

**Table 2:** Tests to assess the net-state-of immunity, and the results infection (immunosuppression) and rejection (immune activation) risk.

TEST	INFECTION/IMMUNOSUPPRESSION RISK	REJECTION/IMMUNE ACTIVATION RISK
**Donor Specific antibody**	Increased background risk with DSA+ (immunosuppression burden/previous rejection)	Positive DSA (HLA and non-HLA) High MFI >4000, rising titers, type (DQ) (154–156, 161, 269).
**Drug levels**	High IS levels: Particularly when Tacrolimus level >8ng/ml (141, 142, 144), MMF.AUC (>60mg/L.h) (142, 146, 147)	Low drug level (Tacrolimus <5ng/ml, MMF.AUC <40mg/L.h) (144, 146) & high Tac variability (140, 143)
**Cell counts**	Leukopenia, Neutropenia, Lymphopenia (73)	Leukocytosis/lymphocytosis
**Immunoglobulins**	Hypogammaglobulinemia (particularly IgG <600 mg/dL) (97–99)	
**Lymphocyte subsets**	Impaired NK cell function and low count (<30 cells/µL) (90, 91) Low CD4+ count (<350 cells/µL), higher risk of OI with (<200 cells/µL) (81, 83). Low CD4/CD8 ratio <1, high proportion CD8+ (>90^th^ centile) Increase in exhausted T-cells* (inhibitory surface markers) (63, 73, 119, 219).	Elevated T-cell (CD8+, CD4+ (>497cells/µL), High effector T cell/T-reg ratio (82, 85, 86, 270) Increased total NK cell count, low CD56+^dim^ NK cells (76, 90, 94, 95).
**Complement**	Low complements: C3 <80 mg/dL, C4 <18 mg/dL (73, 102, 103, 119). Low MBL (104, 119, 271)	
**Soluble CD30**	Low (<90 U/mL) (105, 107)	High (74, 107, 108, 110)
**Cell mediated immune assay**	Negative virus specific CMI, particularly CMV-CMI (129–132, 134, 135)	Positive/High alloreactive B & T-cell ELISPOT assay (IFN- γ, IL-2) (136–139).
**CD4+ iATP assay**	Low ImmuKNOW assay results, (<225ng/mL) (111–113)	High ImmuKNOW assay result (unclear evidence) (113–115) - higher risk if ATP >280 ng/ml (115)
**Viral loads**	Higher counts = greater degree of immunosuppression (14, 120) - BK (particularly >10,000 copies/ml) (121) - Teno-torque virus (122, 123, 125–128, 272)	Suppressed/undetectable viral loads
**Donor derived cell free DNA**	>1% with allograft infection i.e., pyelonephritis of renal allograft, pneumonia in lung transplant recipients (173, 174).	Elevated dd-cfDNA (>1%), in absence of other causes of graft injury (i.e. infection) (168–171). Improved discriminatory performance when combined with clinical, molecular, DSA and protein markers (e.g. gene expression profiling, DSA, urinary chemokines) (187, 198, 263).
**Urinary chemokines**	Elevated in BK Nephropathy and UTI in KTRs (197, 198).	CXCL10, CXCL11 (clinical and subclinical rejection screening) (196, 197, 200, 202)
**Molecular diagnostics**	Gene transcripts associated with infection (129, 207, 208, 273). miRNA associated with infection (230–233, 235–237)	Transcripts/gene-sets associated with subclinical or acute rejection (MDMx, AlloMAP, TruGRAF, kSORT) (134, 210, 212–215, 217, 221, 223–225, 264, 274) miRNA associated with rejection (226, 227, 240, 243, 248, 251, 267) Exomes associated with rejection (253–261)

DSA, Donor specific antibody; HLA, human leukocyte antigen; IS, immunosuppression; MIF, Mean Fluorescence Index; NK, Natural Killer; MMF. AUC, Mycophenolate area-under-curve; MDMx, molecular microscope diagnostic system; miRNA, microribonucleic acid.

If concerns regarding accessibility and affordability are circumvented, validated biomarkers should ideally be performed at regular intervals during high-risk periods for rejection and infection, particularly in transplant recipients with high immunologic risk. The first-year post-transplant carries significant risks for both infection and rejection. Monitoring during this period with 1-3 monthly biomarkers may allow for early detection of graft injury. Identifying the appropriate clinical cause of graft injury requires integrating the biomarkers with the clinical picture, infection-rejection risk assessments (e.g., immunological risk, donor/recipient serological match, chemoprophylaxis, previous rejection), and relevant investigations (viral serology, CMV-CMI, specimen cultures, inflammatory markers, biopsy).

Biomarkers could enhance pre-transplant risk and immunocompatibility assessments and allow for personalization of induction and maintenance immunosuppression and post-transplant monitoring. A positive ELISPOT (IFN-γ) identified individuals with donor-reactive memory and/or effector B and T-cells who were at a higher risk of rejection and may require a more potent induction regimen (e.g., Anti-thymocyte globulin) (268).

Other clinically useful time points include following anti-rejection therapy, completion of antimicrobial prophylaxis or infection therapies, and immunosuppression adjustments (e.g., escalation following rejection or modulation following infections such as BK/CMV).

## 6 Future direction and conclusion

Understanding the transplant recipient’s net state of immunity and balancing the infection-rejection risk axis is a constant challenge for clinicians. The cause-and-effect relationship between infection and rejection is complicated and intricate, with several shared risk factors. Heightening immunosuppression reduces the risk of rejection, albeit with increased risk of rejection and short and long-term immunosuppression-related toxicities. Accurately identifying transplant recipients with allograft tolerance remains elusive. Developing effective measures to identify graft tolerance will enable the safe reduction of immunosuppression and its attendant complications.

Conventional markers of allograft dysfunction, such as eGFR and proteinuria, are not sufficiently sensitive nor specific for timely diagnosis of allograft rejection, with their perturbations often trailing behind immunological injury. Biomarkers, particularly when integrated with clinical, immunological, and serological parameters, could help bridge this gap. Several commercially available tests include but are not limited to: Luminex DSA detection assays (anti-HLA, and non-HLA antibodies); dd-cfDNA quantification (Allosure^®^); urinary chemokine measurement; molecular diagnostics (Allomap^®^, TruGraf, kSORT assay) and immune profiling (ELIspot, ImmuKNOW).

Despite considerable focus and research efforts devoted to identifying non-invasive biomarkers of graft health, most currently fall short of clinical implementation. The complex and evolving immunological changes associated with rejection may not be accurately reflected by a single biomarker alone. Many of the described biomarkers do not consistently demonstrate both high sensitivity, specificity, and favorable predictive values. Additionally, the lack of standardization and validation of commercial assays and diagnostic thresholds with respect to different biological tissue types, clinical phenotypes and patient populations, significantly limits the universal adoption of several emerging biomarkers into routine clinical practice. The significant costs and lack of commercial availability further hinder the transition of some biomarkers from bench to bedside.

Dd-cfDNA and urinary chemokines are the most promising biomarkers that have garnered significant interest with increasing commercial availability. They are now incorporated into several international clinical guidelines for the assessment of allograft dysfunction (185). Dd-cfDNA is particularly advantageous as it is applicable to all SOT and its clinical use has gathered considerable momentum, particularly in the United States (191, 194). Several large clinical trials have been conducted to validate the predictive and diagnostic test characteristics of both dd-cfDNA and urinary chemokines, particularly for the detection or exclusion of AMR (170, 191, 275). Their high NPV makes them especially effective for excluding allograft injury and minimizing unnecessary biopsies (275). However, their poor specificity for rejection diagnosis and inability to distinguish rejection phenotypes may dampen the enthusiasm for widespread clinical uptake. In addition to their role in rejection diagnosis, both dd-cfDNA and urinary chemokines have shown potential for use in detecting and monitoring graft infections such as CMV, BK nephropathy, UTI and pneumonia (197, 199).

Emerging evidence suggests that dd-cfDNA and urinary chemokines may be beneficial in excluding subclinical rejection in those with stable graft function and for assessing immunological injury resolution following anti-rejection therapy (170, 191). Despite promising evidence, the utility of biomarkers in detecting subclinical allograft injury has yet to be verified. Prospective clinical trials are currently underway to clarify their role in this context. Temporal variations of these biomarkers, pre-test risk modifiers, and confounding factors need to be carefully considered when interpreting the results. The incorporation of dd-cfDNA and urinary chemokines in the proper clinical context in conjunction with other predictive and diagnostic immune and non-immune markers could further strengthen the infection-rejection risk stratification algorithm.

Molecular profiling (gene expression, miRNA, exomes) methods, such as the Molecular Microscope Diagnostics system, is also making significant ground and could drastically advance transplant diagnostics. Rejection is a complex process involving the graft, circulating immune cells, and secondary/tertiary lymphoid tissue/organs. Molecular changes at all of these levels must be clearly understood to better appreciate the underlying immunological changes underpinning rejection. Furthermore, identified rejection-specific gene signatures need to be reproduced and validated in large, prospective trials. If this is achieved, molecular diagnostics may eventually enable clinicians to refine the diagnosis of rejection phenotypes and provide insights into the intragraft mechanistic impacts of rejection and anti-rejection therapies.

Biomarkers would be particularly beneficial in clinical scenarios associated with high infection and/or rejection risk, and where currently available risk assessment strategies are imprecise. While allograft biopsies are integral for definitive rejection diagnosis, they are invasive and carry organ-specific risks. Incorporating immunological, serological, and clinical parameters with minimally invasive biomarkers could revolutionize transplant recipient care by allowing serial surveillance of allograft health, enhanced risk stratification of infection and rejection, and personalization of therapeutic and diagnostic decisions. The overarching goal is to detect immunological injury at its earliest stage when therapeutic interventions are most likely to be effective. Developing biomarkers that accurately identify subclinical rejection could substantially advance this goal and significantly enhance both allograft and patient survival.

## References

[1] Ying T Shi B Kelly PJ Pilmore H Clayton PA Chadban SJ. Death after kidney transplantation: an analysis by era and time post-transplant. J Am Soc Nephrol. (2020) 31:2887–99. doi: 10.1681/ASN.2020050566 PMC7790214 32908001

[2] Cainelli F Vento S. Infections and solid organ transplant rejection: a cause-and-effect relationship? Lancet Infect Dis. (2002) 2:539–49. doi: 10.1016/s1473-3099(02)00370-5 12206970

[3] Martin-Gandul C Mueller NJ Pascual M Manuel O. The impact of infection on chronic allograft dysfunction and allograft survival after solid organ transplantation. Am J Transplant. (2015) 15:3024–40. doi: 10.1111/ajt.13486 26474168

[4] Yang B Ye Q Huang C Ding X. Impact of infection-related immunosuppressant reduction on kidney transplant outcomes: A retrospective study considering the temporal dynamics of immunosuppressive requirements. Transpl Int. (2023) 36:11802. doi: 10.3389/ti.2023.11802 38058354 PMC10697076

[5] Shen CL Wu BS Lien TJ Yang AH Yang CY. BK polyomavirus nephropathy in kidney transplantation: balancing rejection and infection. Viruses. (2021) 13:487. doi: 10.3390/v13030487 33809472 PMC7998398

[6] Chen YX Li R Gu L Xu KY Liu YZ Zhang RW. Risk factors and etiology of repeat infection in kidney transplant recipients. Med (Baltimore). (2019) 98:e17312. doi: 10.1097/MD.0000000000017312 PMC6756622 31568017

[7] De Keyzer K Van Laecke S Peeters P Vanholder R. Human cytomegalovirus and kidney transplantation: a clinician’s update. Am J Kidney Dis. (2011) 58:118–26. doi: 10.1053/j.ajkd.2011.04.010 21684438

[8] Halskov ACL Dagnaes-Hansen J Stroomberg HV Sorensen SS Roder A. Incidence of and risk factors for recurrent urinary tract infections in renal transplant recipients. Eur Urol Open Sci. (2023) 52:115–22. doi: 10.1016/j.euros.2023.04.001 PMC10240509 37284043

[9] Higdon LE Tan JC Maltzman JS. Infection, rejection, and the connection. Transplantation. (2023) 107:584–95. doi: 10.1097/TP.0000000000004297 PMC9968362 36017937

[10] Khalil MAM Khalil MAU Khan TFT Tan J. Drug-induced hematological cytopenia in kidney transplantation and the challenges it poses for kidney transplant physicians. J Transplant. (2018) 2018:9429265. doi: 10.1155/2018/9429265 30155279 PMC6093016

[11] Stewart AG Kotton CN. What’s new: updates on cytomegalovirus in solid organ transplantation. Transplantation. (2024) 108:884–97. doi: 10.1097/TP.0000000000004855 37899366

[12] Furness PN Taub N Assmann KJ Banfi G Cosyns JP Dorman AM. International variation in histologic grading is large, and persistent feedback does not improve reproducibility. Am J Surg Pathol. (2003) 27:805–10. doi: 10.1097/00000478-200306000-00012 12766585

[13] Furness PN Taub N. Convergence of European Renal Transplant Pathology Assessment Procedures P. International variation in the interpretation of renal transplant biopsies: report of the CERTPAP Project. Kidney Int. (2001) 60:1998–2012. doi: 10.1046/j.1523-1755.2001.00030.x 11703620

[14] Roberts MB Fishman JA. Immunosuppressive agents and infectious risk in transplantation: managing the “Net state of immunosuppression. Clin Infect Dis. (2021) 73:e1302–e17. doi: 10.1093/cid/ciaa1189 PMC8561260 32803228

[15] Agrawal A Ison MG Danziger-Isakov L. Long-term infectious complications of kidney transplantation. Clin J Am Soc Nephrol. (2022) 17:286–95. doi: 10.2215/CJN.15971020 PMC8823942 33879502

[16] Fishman JA. Infection in organ transplantation. Am J Transplant. (2017) 17:856–79. doi: 10.1111/ajt.14208 28117944

[17] Hart A Singh D Brown SJ Wang JH Kasiske BL. Incidence, risk factors, treatment, and consequences of antibody-mediated kidney transplant rejection: A systematic review. Clin Transplant. (2021) 35:e14320. doi: 10.1111/ctr.14320 33864724

[18] Oweira H Ramouz A Ghamarnejad O Khajeh E Ali-Hasan-Al-Saegh S Nikbakhsh R. Risk factors of rejection in renal transplant recipients: A narrative review. J Clin Med. (2022) 11:1392. doi: 10.3390/jcm11051392 35268482 PMC8911293

[19] Cippa PE Schiesser M Ekberg H van Gelder T Mueller NJ Cao CA. Risk stratification for rejection and infection after kidney transplantation. Clin J Am Soc Nephrol. (2015) 10:2213–20. doi: 10.2215/CJN.01790215 PMC4670759 26430088

[20] D’Orsogna L van den Heuvel H van Kooten C Heidt S Claas FHJ. Infectious pathogens may trigger specific allo-HLA reactivity via multiple mechanisms. Immunogenetics. (2017) 69:631–41. doi: 10.1007/s00251-017-0989-3 PMC5537314 28718002

[21] Johansson I Martensson G Nystrom U Nasic S Andersson R. Lower incidence of CMV infection and acute rejections with valganciclovir prophylaxis in lung transplant recipients. BMC Infect Dis. (2013) 13:582. doi: 10.1186/1471-2334-13-582 24325216 PMC3878887

[22] Razonable RR Humar A. Cytomegalovirus in solid organ transplant recipients-Guidelines of the American Society of Transplantation Infectious Diseases Community of Practice. Clin Transplant. (2019) 33:e13512. doi: 10.1111/ctr.13512 30817026

[23] Ruenroengbun N Sapankaew T Chaiyakittisopon K Phoompoung P Ngamprasertchai T. Efficacy and safety of antiviral agents in preventing allograft rejection following CMV prophylaxis in high-risk kidney transplantation: A systematic review and network meta-analysis of randomized controlled trials. Front Cell Infect Microbiol. (2022) 12:865735. doi: 10.3389/fcimb.2022.865735 35433502 PMC9010655

[24] Slifkin M Ruthazer R Freeman R Bloom J Fitzmaurice S Fairchild R. Impact of cytomegalovirus prophylaxis on rejection following orthotopic liver transplantation. Liver Transpl. (2005) 11:1597–602. doi: 10.1002/(ISSN)1527-6473 16315314

[25] Karahan GE Claas FHJ Heidt S. Heterologous immunity of virus-specific T cells leading to alloreactivity: possible implications for solid organ transplantation. Viruses. (2021) 13:2359. doi: 10.3390/v13122359 34960628 PMC8706157

[26] Adams AB Williams MA Jones TR Shirasugi N Durham MM Kaech SM. Heterologous immunity provides a potent barrier to transplantation tolerance. J Clin Invest. (2003) 111:1887–95. doi: 10.1172/JCI200317477 PMC161424 12813024

[27] Kaminski H Fishman JA. The cell biology of cytomegalovirus: implications for transplantation. Am J Transplant. (2016) 16:2254–69. doi: 10.1111/ajt.13791 26991039

[28] Marks WH Mamode N Montgomery RA Stegall MD Ratner LE Cornell LD. Safety and efficacy of eculizumab in the prevention of antibody-mediated rejection in living-donor kidney transplant recipients requiring desensitization therapy: A randomized trial. Am J Transplant. (2019) 19:2876–88. doi: 10.1111/ajt.15364 PMC6790671 30887675

[29] Mohamed N Aggarwal V Cole E John R. Histopathologic detection of rejection in acute allograft pyelonephritis. Transplantation. (2012) 94:e46–7. doi: 10.1097/TP.0b013e318265c4b8 23038631

[30] Couzi L Pitard V Moreau JF Merville P Dechanet-Merville J. Direct and Indirect Effects of Cytomegalovirus-Induced gammadelta T Cells after Kidney Transplantation. Front Immunol. (2015) 6:3. doi: 10.3389/fimmu.2015.00003 25653652 PMC4301015

[31] Masset C Gautier-Vargas G Cantarovich D Ville S Dantal J Delbos F. Occurrence of *de novo* donor-specific antibodies after COVID-19 in kidney transplant recipients is low despite immunosuppression modulation. Kidney Int Rep. (2022) 7:983–92. doi: 10.1016/j.ekir.2022.01.1072 PMC8818557 35155848

[32] Patel SJ Kuten SA Knight RJ Graviss EA Nguyen D Gaber AO. Incidence and factors associated with *de novo* DSA after BK viremia in renal transplant recipients. Clin Transpl. (2016) 32:103–9. 28564527

[33] Vasquez-Jimenez E Moguel-Gonzalez B Soto-Abraham V Flores-Gama C. Risk of acute rejection in kidney transplant recipients after COVID-19. J Nephrol. (2022) 35:367–9. doi: 10.1007/s40620-021-01192-x PMC8596849 34787799

[34] Shah SZ Abdelmoneim Y Pham SM Elrefaei M. Association between SARS-CoV-2 infection and *de novo* HLA donor specific antibody production in lung transplant recipients: Single-center study. Hum Immunol. (2022) 83:749–54. doi: 10.1016/j.humimm.2022.07.007 PMC9376302 35987702

[35] Foley B Cooley S Verneris MR Curtsinger J Luo X Waller EK. Human cytomegalovirus (CMV)-induced memory-like NKG2C(+) NK cells are transplantable and expand in *vivo* in response to recipient CMV antigen. J Immunol. (2012) 189:5082–8. doi: 10.4049/jimmunol.1201964 PMC3490031 23077239

[36] Pickering H Sen S Arakawa-Hoyt J Ishiyama K Sun Y Parmar R. NK and CD8+ T cell phenotypes predict onset and control of CMV viremia after kidney transplant. JCI Insight. (2021) 6:e153175. doi: 10.1172/jci.insight.153175 34609965 PMC8663544

[37] Borchers AT Perez R Kaysen G Ansari AA Gershwin ME. Role of cytomegalovirus infection in allograft rejection: a review of possible mechanisms. Transpl Immunol. (1999) 7:75–82. doi: 10.1016/S0966-3274(99)80023-9 10544437

[38] Koskinen PK. The association of the induction of vascular cell adhesion molecule-1 with cytomegalovirus antigenemia in human heart allografts. Transplantation. (1993) 56:1103–8. doi: 10.1097/00007890-199311000-00011 7504341

[39] Yilmaz S Koskinen PK Kallio E Bruggeman CA Hayry PJ Lemstrom KB. Cytomegalovirus infection-enhanced chronic kidney allograft rejection is linked with intercellular adhesion molecule-1 expression. Kidney Int. (1996) 50:526–37. doi: 10.1038/ki.1996.345 8840282

[40] Ustinov J Loginov R Bruggeman C van der Meide P Hayry P Lautenschlager I. Direct induction of class II antigens by cytomegalovirus in rat heart endothelial cells. Transplant Proc. (1993) 25:1143–4. 8382852

[41] Nellore A Fishman JA. The microbiome, systemic immune function, and allotransplantation. Clin Microbiol Rev. (2016) 29:191–9. doi: 10.1128/CMR.00063-15 PMC4771220 26656674

[42] Higdon LE Gustafson CE Ji X Sahoo MK Pinsky BA Margulies KB. Association of premature immune aging and cytomegalovirus after solid organ transplant. Front Immunol. (2021) 12:661551. doi: 10.3389/fimmu.2021.661551 34122420 PMC8190404

[43] Martins PN Tullius SG Markmann JF. Immunosenescence and immune response in organ transplantation. Int Rev Immunol. (2014) 33:162–73. doi: 10.3109/08830185.2013.829469 PMC5497513 24127845

[44] Manook M Koeser L Ahmed Z Robb M Johnson R Shaw O. Post-listing survival for highly sensitised patients on the UK kidney transplant waiting list: a matched cohort analysis. Lancet. (2017) 389:727–34. doi: 10.1016/S0140-6736(16)31595-1 28065559

[45] Selin LK Cornberg M Brehm MA Kim SK Calcagno C Ghersi D. CD8 memory T cells: cross-reactivity and heterologous immunity. Semin Immunol. (2004) 16:335–47. doi: 10.1016/j.smim.2004.08.014 PMC7128110 15528078

[46] Yurochko AD Huang ES. Human cytomegalovirus binding to human monocytes induces immunoregulatory gene expression. J Immunol. (1999) 162:4806–16. doi: 10.4049/jimmunol.162.8.4806 10202024

[47] Ahn R Schaenman J Qian Z Pickering H Groysberg V Rossetti M. Acute and chronic changes in gene expression after CMV DNAemia in kidney transplant recipients. Front Immunol. (2021) 12:750659. doi: 10.3389/fimmu.2021.750659 34867983 PMC8634678

[48] Denecke C Tullius SG. Innate and adaptive immune responses subsequent to ischemia-reperfusion injury in the kidney. Prog Urol. (2014) 24 Suppl 1:S13–9. doi: 10.1016/S1166-7087(14)70058-2 24950927

[49] Lumbreras C Manuel O Len O ten Berge IJ Sgarabotto D Hirsch HH. Cytomegalovirus infection in solid organ transplant recipients. Clin Microbiol Infect. (2014) 20 Suppl 7:19–26. doi: 10.1111/1469-0691.12594 26451404

[50] Nieuwenhuijs-Moeke GJ Pischke SE Berger SP Sanders JSF Pol RA Struys M. Ischemia and reperfusion injury in kidney transplantation: relevant mechanisms in injury and repair. J Clin Med. (2020) 9:253. doi: 10.3390/jcm9010253 31963521 PMC7019324

[51] Zhao H Alam A Soo AP George AJT Ma D. Ischemia-reperfusion injury reduces long term renal graft survival: mechanism and beyond. EBioMedicine. (2018) 28:31–42. doi: 10.1016/j.ebiom.2018.01.025 29398595 PMC5835570

[52] Basile DP. The endothelial cell in ischemic acute kidney injury: implications for acute and chronic function. Kidney Int. (2007) 72:151–6. doi: 10.1038/sj.ki.5002312 17495858

[53] Dolmatova EV Wang K Mandavilli R Griendling KK. The effects of sepsis on endothelium and clinical implications. Cardiovasc Res. (2021) 117:60–73. doi: 10.1093/cvr/cvaa070 32215570 PMC7810126

[54] Hall AV Jevnikar AM. Significance of endothelial cell survival programs for renal transplantation. Am J Kidney Dis. (2003) 41:1140–54. doi: 10.1016/S0272-6386(03)00345-7 12776265

[55] Crombie JL LaCasce AS. Epstein barr virus associated B-cell lymphomas and iatrogenic lymphoproliferative disorders. Front Oncol. (2019) 9:109. doi: 10.3389/fonc.2019.00109 30899698 PMC6416204

[56] Nijland ML Kersten MJ Pals ST Bemelman FJ Ten Berge IJ. Epstein-barr virus-positive posttransplant lymphoproliferative disease after solid organ transplantation: pathogenesis, clinical manifestations, diagnosis, and management. Transplant Direct. (2016) 2:e48. doi: 10.1097/TXD.0000000000000557 27500242 PMC4946499

[57] Fernandez-Ruiz M Lopez-Medrano F Allende LM Andres A Garcia-Reyne A Lumbreras C. Kinetics of peripheral blood lymphocyte subpopulations predicts the occurrence of opportunistic infection after kidney transplantation. Transpl Int. (2014) 27:674–85. doi: 10.1111/tri.2014.27.issue-7 24650360

[58] Gardiner BJ Nierenberg NE Chow JK Ruthazer R Kent DM Snydman DR. Absolute lymphocyte count: A predictor of recurrent cytomegalovirus disease in solid organ transplant recipients. Clin Infect Dis. (2018) 67:1395–402. doi: 10.1093/cid/ciy295 PMC6927884 29635432

[59] Warny M Helby J Nordestgaard BG Birgens H Bojesen SE. Lymphopenia and risk of infection and infection-related death in 98,344 individuals from a prospective Danish population-based study. PloS Med. (2018) 15:e1002685. doi: 10.1371/journal.pmed.1002685 30383787 PMC6211632

[60] Zafrani L Truffaut L Kreis H Etienne D Rafat C Lechaton S. Incidence, risk factors and clinical consequences of neutropenia following kidney transplantation: a retrospective study. Am J Transplant. (2009) 9:1816–25. doi: 10.1111/j.1600-6143.2009.02699.x 19538494

[61] Kotton CN Kumar D Caliendo AM Huprikar S Chou S Danziger-Isakov L. The third international consensus guidelines on the management of cytomegalovirus in solid-organ transplantation. Transplantation. (2018) 102:900–31. doi: 10.1097/TP.0000000000002191 29596116

[62] British Transplantation Society (BTS). UK guideline on prevention and management of cytomegalovirus (cmv) infection and disease following solid organ transplantation, British transplantation Society. (2022). Available at: https://bts.org.uk/uk-guideline-on-prevention-and-management-of-cytomegalovirus-cmv-infection-and-disease-following-solid-organ-transplantation/. (Accessed January 24, 2024).

[63] Crepin T Legendre M Carron C Vachey C Courivaud C Rebibou JM. Uraemia-induced immune senescence and clinical outcomes in chronic kidney disease patients. Nephrol Dial Transplant. (2020) 35:624–32. doi: 10.1093/ndt/gfy276 30202981

[64] Stangou MJ Fylaktou A Ivanova-Shivarova MI Theodorou I. Editorial: immunosenescence and immunoexhaustion in chronic kidney disease and renal transplantation. Front Med (Lausanne). (2022) 9:874581. doi: 10.3389/fmed.2022.874581 35479944 PMC9037092

[65] Sanders JF Bemelman FJ Messchendorp AL Baan CC van Baarle D van Binnendijk R. The RECOVAC immune-response study: the immunogenicity, tolerability, and safety of COVID-19 vaccination in patients with chronic kidney disease, on dialysis, or living with a kidney transplant. Transplantation. (2022) 106:821–34. doi: 10.1097/TP.0000000000003983 PMC8942603 34753894

[66] Maggiore U Abramowicz D Crespo M Mariat C Mjoen G Peruzzi L. How should I manage immunosuppression in a kidney transplant patient with COVID-19? An ERA-EDTA DESCARTES expert opinion. Nephrol Dial Transplant. (2020) 35:899–904. doi: 10.1093/ndt/gfaa130 32441741 PMC7313836

[67] Yahav D Sulimani O Green H Margalit I Ben-Zvi H Mor E. Immunosuppression reduction in kidney transplant recipients during bacterial infection-A retrospective study. Clin Transplant. (2019) 33:e13707. doi: 10.1111/ctr.v33.10 31494965

[68] Adam B Afzali B Dominy KM Chapman E Gill R Hidalgo LG. Multiplexed color-coded probe-based gene expression assessment for clinical molecular diagnostics in formalin-fixed paraffin-embedded human renal allograft tissue. Clin Transplant. (2016) 30:295–305. doi: 10.1111/ctr.2016.30.issue-3 26729350

[69] Bowman LJ Brueckner AJ Doligalski CT. The role of mTOR inhibitors in the management of viral infections: A review of current literature. Transplantation. (2018) 102:S50–S9. doi: 10.1097/TP.0000000000001777 29369973

[70] Dzankic PJ Partridge L. Clinical trials of mTOR inhibitors to boost immunity to viral infection in older adults. Lancet Healthy Longev. (2021) 2:e232–e3. doi: 10.1016/S2666-7568(21)00090-8 PMC8102037 33977282

[71] Karam BS Morris RS Bramante CT Puskarich M Zolfaghari EJ Lotfi-Emran S. mTOR inhibition in COVID-19: A commentary and review of efficacy in RNA viruses. J Med Virol. (2021) 93:1843–6. doi: 10.1002/jmv.26728 PMC8159020 33314219

[72] Pinchera B Spirito L Buonomo AR Foggia M Carrano R Salemi F. mTOR inhibitor use is associated with a favorable outcome of COVID-19 in patients of kidney transplant: results of a retrospective study. Front Med (Lausanne). (2022) 9:852973. doi: 10.3389/fmed.2022.852973 35801204 PMC9254357

[73] Dendle C Mulley WR Holdsworth S. Can immune biomarkers predict infections in solid organ transplant recipients? A review of current evidence. Transplant Rev (Orlando). (2019) 33:87–98. doi: 10.1016/j.trre.2018.10.001 30551846

[74] Mirzakhani M Shahbazi M Akbari R Dedinska I Nemati E Mohammadnia-Afrouzi M. Soluble CD30, the immune response, and acute rejection in human kidney transplantation: A systematic review and meta-analysis. Front Immunol. (2020) 11:295. doi: 10.3389/fimmu.2020.00295 32256486 PMC7093023

[75] Rodrigo E Lopez-Hoyos M Corral M Fabrega E Fernandez-Fresnedo G San Segundo D. ImmuKnow as a diagnostic tool for predicting infection and acute rejection in adult liver transplant recipients: a systematic review and meta-analysis. Liver Transpl. (2012) 18:1245–53. doi: 10.1002/lt.v18.10 22740321

[76] Pontrelli P Rascio F Castellano G Grandaliano G Gesualdo L Stallone G. The role of natural killer cells in the immune response in kidney transplantation. Front Immunol. (2020) 11:1454. doi: 10.3389/fimmu.2020.01454 32793200 PMC7390843

[77] Kitajima T Rajendran L Lisznyai E Lu M Shamaa T Ivanics T. Lymphopenia at the time of transplant is associated with short-term mortality after deceased donor liver transplantation. Am J Transplant. (2023) 23:248–56. doi: 10.1016/j.ajt.2022.11.004 36804132

[78] Perry WA Paulus JK Price LL Snydman DR Chow JK. Association between lymphopenia at 1 month posttransplant and infectious outcomes or death in heart transplant recipients. Clin Infect Dis. (2021) 73:e3797–e803. doi: 10.1093/cid/ciaa1800 PMC8664493 33279963

[79] Schoeberl AK Zuckermann A Kaider A Aliabadi-Zuckermann A Uyanik-Uenal K Laufer G. Absolute lymphocyte count as a marker for cytomegalovirus infection after heart transplantation. Transplantation. (2023) 107:748–52. doi: 10.1097/TP.0000000000004360 36228318

[80] Dujardin A Lorent M Foucher Y Legendre C Kerleau C Brouard S. Time-dependent lymphocyte count after transplantation is associated with higher risk of graft failure and death. Kidney Int. (2021) 99:1189–201. doi: 10.1016/j.kint.2020.08.010 32891605

[81] Calarota SA Chiesa A De Silvestri A Morosini M Oggionni T Marone P. T-lymphocyte subsets in lung transplant recipients: association between nadir CD4 T-cell count and viral infections after transplantation. J Clin Virol. (2015) 69:110–6. doi: 10.1016/j.jcv.2015.06.078 PMC7106454 26209391

[82] Abdullah EL Jalalonmuhali M Ng KP Jamaluddin FA Lim SK. The role of lymphocyte subset in predicting allograft rejections in kidney transplant recipients. Transplant Proc. (2022) 54:312–9. doi: 10.1016/j.transproceed.2022.01.009 35246329

[83] Calarota SA Zelini P De Silvestri A Chiesa A Comolli G Sarchi E. Kinetics of T-lymphocyte subsets and posttransplant opportunistic infections in heart and kidney transplant recipients. Transplantation. (2012) 93:112–9. doi: 10.1097/TP.0b013e318239e90c 22134368

[84] Meesing A Abraham RS Razonable RR. Clinical correlation of cytomegalovirus infection with CMV-specific CD8+ T-cell immune competence score and lymphocyte subsets in solid organ transplant recipients. Transplantation. (2019) 103:832–8. doi: 10.1097/TP.0000000000002396 30086091

[85] Bernaldo-de-Quiros E Lopez-Abente J Camino M Gil N Panadero E Lopez-Esteban R. The presence of a marked imbalance between regulatory T cells and effector T cells reveals that tolerance mechanisms could be compromised in heart transplant children. Transplant Direct. (2021) 7:e693. doi: 10.1097/TXD.0000000000001152 33928185 PMC8078462

[86] Taubert R Danger R Londono MC Christakoudi S Martinez-Picola M Rimola A. Hepatic infiltrates in operational tolerant patients after liver transplantation show enrichment of regulatory T cells before proinflammatory genes are downregulated. Am J Transplant. (2016) 16:1285–93. doi: 10.1111/ajt.13617 26603835

[87] Betjes MG Meijers RW de Wit EA Weimar W Litjens NH. Terminally differentiated CD8+ Temra cells are associated with the risk for acute kidney allograft rejection. Transplantation. (2012) 94:63–9. doi: 10.1097/TP.0b013e31825306ff 22691956

[88] Mai HL Degauque N Le Bot S Rimbert M Renaudin K Danger R. Antibody-mediated allograft rejection is associated with an increase in peripheral differentiated CD28-CD8+ T cells - Analyses of a cohort of 1032 kidney transplant recipients. EBioMedicine. (2022) 83:104226. doi: 10.1016/j.ebiom.2022.104226 35988467 PMC9420477

[89] Mou D Espinosa J Lo DJ Kirk AD. CD28 negative T cells: is their loss our gain? Am J Transplant. (2014) 14:2460–6. doi: 10.1111/ajt.12937 PMC4886707 25323029

[90] Dendle C Gan PY Polkinghorne KR Ngui J Stuart RL Kanellis J. Natural killer cell function predicts severe infection in kidney transplant recipients. Am J Transplant. (2019) 19:166–77. doi: 10.1111/ajt.14900 29708649

[91] Fernandez-Ruiz M Silva JT Lopez-Medrano F Allende LM San Juan R Cambra F. Post-transplant monitoring of NK cell counts as a simple approach to predict the occurrence of opportunistic infection in liver transplant recipients. Transpl Infect Dis. (2016) 18:552–65. doi: 10.1111/tid.12564 27260953

[92] Qin R Qin J Li X Xu Z He P Yuan X. Influence of immunosuppressive drugs on natural killer cells in therapeutic drug exposure in liver transplantation. Hepatobil Surg Nutr. (2023) 12:835–53. doi: 10.21037/hbsn-22-438 PMC10727815 38115918

[93] Yagisawa T Tanaka T Miyairi S Tanabe K Dvorina N Yokoyama WM. In the absence of natural killer cell activation donor-specific antibody mediates chronic, but not acute, kidney allograft rejection. Kidney Int. (2019) 95:350–62. doi: 10.1016/j.kint.2018.08.041 PMC6342643 30503624

[94] Sablik KA Litjens NHR Klepper M Betjes MGH. Increased CD16 expression on NK cells is indicative of antibody-dependent cell-mediated cytotoxicity in chronic-active antibody-mediated rejection. Transpl Immunol. (2019) 54:52–8. doi: 10.1016/j.trim.2019.02.005 30794946

[95] Fildes JE Yonan N Tunstall K Walker AH Griffiths-Davies L Bishop P. Natural killer cells in peripheral blood and lung tissue are associated with chronic rejection after lung transplantation. J Heart Lung Transplant. (2008) 27:203–7. doi: 10.1016/j.healun.2007.11.571 18267228

[96] Song S Zhi Y Tian G Sun X Chen Y Qiu W. Immature and activated phenotype of blood NK cells is associated with acute rejection in adult liver transplant. Liver Transpl. (2023) 29:836–48. doi: 10.1097/LVT.0000000000000139 37002601

[97] Fernandez-Ruiz M Lopez-Medrano F Varela-Pena P Lora-Pablos D Garcia-Reyne A Gonzalez E. Monitoring of immunoglobulin levels identifies kidney transplant recipients at high risk of infection. Am J Transplant. (2012) 12:2763–73. doi: 10.1111/j.1600-6143.2012.04192.x 22823002

[98] Florescu DF Kalil AC Qiu F Schmidt CM Sandkovsky U. What is the impact of hypogammaglobulinemia on the rate of infections and survival in solid organ transplantation? A meta-analysis. Am J Transplant. (2013) 13:2601–10. doi: 10.1111/ajt.12401 23919557

[99] Jo EA Min S Jo AJ Han A Ha J Song EY. The time-dependent changes in serum immunoglobulin after kidney transplantation and its association with infection. Front Immunol. (2024) 15:1374535. doi: 10.3389/fimmu.2024.1374535 38707898 PMC11066164

[100] Sarmiento E Arraya M Jaramillo M Diez P Fernandez-Yanez J Palomo J. Intravenous immunoglobulin as an intervention strategy of risk factor modification for prevention of severe infection in heart transplantation. Clin Exp Immunol. (2014) 178 Suppl 1:156–8. doi: 10.1111/cei.12552 PMC4285532 25546803

[101] Jordan SC Toyoda M Kahwaji J Vo AA. Clinical aspects of intravenous immunoglobulin use in solid organ transplant recipients. Am J Transplant. (2011) 11:196–202. doi: 10.1111/j.1600-6143.2010.03400.x 21219579

[102] Fernandez-Ruiz M Lopez-Medrano F Varela-Pena P Morales JM Garcia-Reyne A San Juan R. Hypocomplementemia in kidney transplant recipients: impact on the risk of infectious complications. Am J Transplant. (2013) 13:685–94. doi: 10.1111/ajt.12055 23311502

[103] Carbone J Micheloud D Salcedo M Rincon D Banares R Clemente G. Humoral and cellular immune monitoring might be useful to identify liver transplant recipients at risk for development of infection. Transpl Infect Dis. (2008) 10:396–402. doi: 10.1111/j.1399-3062.2008.00329.x 18657086

[104] Lombardo-Quezada J Sanclemente G Colmenero J Espanol-Rego M Arias MT Ruiz P. Mannose-binding lectin-deficient donors increase the risk of bacterial infection and bacterial infection-related mortality after liver transplantation. Am J Transplant. (2018) 18:197–206. doi: 10.1111/ajt.14408 28649744

[105] Spiridon C Hunt J Mack M Rosenthal J Anderson A Eichhorn E. Evaluation of soluble CD30 as an immunologic marker in heart transplant recipients. Transplant Proc. (2006) 38:3689–91. doi: 10.1016/j.transproceed.2006.10.088 17175368

[106] Fernandez-Ruiz M Parra P Lopez-Medrano F Ruiz-Merlo T Gonzalez E Polanco N. Serum sCD30: A promising biomarker for predicting the risk of bacterial infection after kidney transplantation. Transpl Infect Dis. (2017) 19. doi: 10.1111/tid.12668 28122147

[107] Wang D Wu WZ Chen JH Yang SL Wang QH Zeng ZX. Pre-transplant soluble CD30 level as a predictor of not only acute rejection and graft loss but pneumonia in renal transplant recipients. Transpl Immunol. (2010) 22:115–20. doi: 10.1016/j.trim.2009.12.004 20036333

[108] Wang D Wu G Chen J Yu Z Wu W Yang S. Predicting renal graft failure by sCD30 levels and *de novo* HLA antibodies at 1year post-transplantation. Transpl Immunol. (2012) 26:235–9. doi: 10.1016/j.trim.2012.03.002 22446727

[109] Chen Y Tai Q Hong S Kong Y Shang Y Liang W. Pretransplantation soluble CD30 level as a predictor of acute rejection in kidney transplantation: a meta-analysis. Transplantation. (2012) 94:911–8. doi: 10.1097/TP.0b013e31826784ad 23052636

[110] de Holanda MI Matuck T de Carvalho D Domingues E Curvo R Glasberg DS. Soluble CD30, acute rejection, and graft survival: pre- and 6-month post-transplant determinations-when is the best time to measure? Transplant Proc. (2018) 50:728–36. doi: 10.1016/j.transproceed.2018.02.025 29661425

[111] Weston MW Rinde-Hoffman D Lopez-Cepero M. Monitoring cell-mediated immunity during immunosuppression reduction in heart transplant recipients with severe systemic infections. Clin Transplant. (2020) 34:e13809. doi: 10.1111/ctr.13809 32003048

[112] Moon HH Kim TS Roh YN Lee S Song S Shin M. Can immune function assay predict infection or recovery? Transplant Proc. (2012) 44:1048–51. doi: 10.1016/j.transproceed.2012.04.001 22564622

[113] Israeli M Ben-Gal T Yaari V Valdman A Matz I Medalion B. Individualized immune monitoring of cardiac transplant recipients by noninvasive longitudinal cellular immunity tests. Transplantation. (2010) 89:968–76. doi: 10.1097/TP.0b013e3181cbabe6 20075792

[114] Fernandez-Ruiz M Kumar D Humar A. Clinical immune-monitoring strategies for predicting infection risk in solid organ transplantation. Clin Transl Immunol. (2014) 3:e12. doi: 10.1038/cti.2014.3 PMC4232060 25505960

[115] Kowalski RJ Post DR Mannon RB Sebastian A Wright HI Sigle G. Assessing relative risks of infection and rejection: a meta-analysis using an immune function assay. Transplantation. (2006) 82:663–8. doi: 10.1097/01.tp.0000234837.02126.70 16969290

[116] Dendle C Polkinghorne KR Mulley WR Gan PY Kanellis J Stuart RL. A simple score can identify kidney transplant recipients at high risk of severe infection over the following 2 years. Transpl Infect Dis. (2019) 21:e13076. doi: 10.1111/tid.2019.21.issue-3 30875147

[117] Crepin T Gaiffe E Courivaud C Roubiou C Laheurte C Moulin B. Pre-transplant end-stage renal disease-related immune risk profile in kidney transplant recipients predicts post-transplant infections. Transpl Infect Dis. (2016) 18:415–22. doi: 10.1111/tid.2016.18.issue-3 27027787

[118] Fernandez-Ruiz M Seron D Alonso A Lora D Hernandez D Gonzalez E. Derivation and external validation of the SIMPLICITY score as a simple immune-based risk score to predict infection in kidney transplant recipients. Kidney Int. (2020) 98:1031–43. doi: 10.1016/j.kint.2020.04.054 32540404

[119] Sarmiento E Navarro J Fernandez-Yanez J Palomo J Munoz P Carbone J. Evaluation of an immunological score to assess the risk of severe infection in heart recipients. Transpl Infect Dis. (2014) 16:802–12. doi: 10.1111/tid.2014.16.issue-5 25179534

[120] De Vlaminck I Khush KK Strehl C Kohli B Luikart H Neff NF. Temporal response of the human virome to immunosuppression and antiviral therapy. Cell. (2013) 155:1178–87. doi: 10.1016/j.cell.2013.10.034 PMC4098717 24267896

[121] Bloom RD Augustine JJ. Beyond the biopsy: monitoring immune status in kidney recipients. Clin J Am Soc Nephrol. (2021) 16:1413–22. doi: 10.2215/CJN.14840920 PMC8729582 34362810

[122] Doberer K Schiemann M Strassl R Haupenthal F Dermuth F Gorzer I. Torque teno virus for risk stratification of graft rejection and infection in kidney transplant recipients-A prospective observational trial. Am J Transplant. (2020) 20:2081–90. doi: 10.1111/ajt.15810 PMC7496119 32034850

[123] Fernandez-Ruiz M. Torque Teno virus load as a surrogate marker for the net state of immunosuppression: The beneficial side of the virome. Am J Transplant. (2020) 20:1963–4. doi: 10.1111/ajt.15872 32189426

[124] Strassl R Doberer K Rasoul-Rockenschaub S Herkner H Gorzer I Klager JP. Torque teno virus for risk stratification of acute biopsy-proven alloreactivity in kidney transplant recipients. J Infect Dis. (2019) 219:1934–9. doi: 10.1093/infdis/jiz039 PMC6534191 30668796

[125] Strassl R Schiemann M Doberer K Gorzer I Puchhammer-Stockl E Eskandary F. Quantification of torque teno virus viremia as a prospective biomarker for infectious disease in kidney allograft recipients. J Infect Dis. (2018) 218:1191–9. doi: 10.1093/infdis/jiy306 PMC6490304 30007341

[126] Kotton CN. Torque teno virus: predictor of infection after solid organ transplant? J Infect Dis. (2018) 218:1185–7. doi: 10.1093/infdis/jiy384 30007368

[127] Maggi F Focosi D Statzu M Bianco G Costa C Macera L. Early post-transplant torquetenovirus viremia predicts cytomegalovirus reactivations in solid organ transplant recipients. Sci Rep. (2018) 8:15490. doi: 10.1038/s41598-018-33909-7 30341363 PMC6195516

[128] Doberer K Haupenthal F Nackenhorst M Bauernfeind F Dermuth F Eigenschink M. Torque teno virus load is associated with subclinical alloreactivity in kidney transplant recipients: A prospective observational trial. Transplantation. (2021) 105:2112–8. doi: 10.1097/TP.0000000000003619 PMC8376270 33587432

[129] Bestard O Kaminski H Couzi L Fernandez-Ruiz M Manuel O. Cytomegalovirus cell-mediated immunity: ready for routine use? Transpl Int. (2023) 36:11963. doi: 10.3389/ti.2023.11963 38020746 PMC10661902

[130] Deborska-Materkowska D Perkowska-Ptasinska A Sadowska A Gozdowska J Ciszek M Serwanska-Swietek M. Diagnostic utility of monitoring cytomegalovirus-specific immunity by QuantiFERON-cytomegalovirus assay in kidney transplant recipients. BMC Infect Dis. (2018) 18:179. doi: 10.1186/s12879-018-3075-z 29661141 PMC5902940

[131] Hall VG Humar A Kumar D. Utility of cytomegalovirus cell-mediated immunity assays in solid organ transplantation. J Clin Microbiol. (2022) 60:e0171621. doi: 10.1128/jcm.01716-21 35543099 PMC9383112

[132] Manuel O Husain S Kumar D Zayas C Mawhorter S Levi ME. Assessment of cytomegalovirus-specific cell-mediated immunity for the prediction of cytomegalovirus disease in high-risk solid-organ transplant recipients: a multicenter cohort study. Clin Infect Dis. (2013) 56:817–24. doi: 10.1093/cid/cis993 23196955

[133] Sester M Leboeuf C Schmidt T Hirsch HH. The “ABC” of virus-specific T cell immunity in solid organ transplantation. Am J Transplant. (2016) 16:1697–706. doi: 10.1111/ajt.13684 26699950

[134] Andreani M Albano L Benzaken S Cassuto E Jeribi A Caramella A. Monitoring of CMV-specific cell-mediated immunity in kidney transplant recipients with a high risk of CMV disease (D+/R-): A case series. Transplant Proc. (2020) 52:204–11. doi: 10.1016/j.transproceed.2019.11.002 31889538

[135] Reusing JO Jr. Agena F Kotton CN Campana G Pierrotti LC David-Neto E. QuantiFERON-CMV as a predictor of CMV events during preemptive therapy in CMV-seropositive kidney transplant recipients. Transplantation. (2024) 108:985–95. doi: 10.1097/TP.0000000000004870 37990351

[136] Augustine JJ Siu DS Clemente MJ Schulak JA Heeger PS Hricik DE. Pre-transplant IFN-gamma ELISPOTs are associated with post-transplant renal function in African American renal transplant recipients. Am J Transplant. (2005) 5:1971–5. doi: 10.1111/j.1600-6143.2005.00958.x 15996247

[137] Hricik DE Rodriguez V Riley J Bryan K Tary-Lehmann M Greenspan N. Enzyme linked immunosorbent spot (ELISPOT) assay for interferon-gamma independently predicts renal function in kidney transplant recipients. Am J Transplant. (2003) 3:878–84. doi: 10.1034/j.1600-6143.2003.00132.x 12814480

[138] Montero N Farouk S Gandolfini I Crespo E Jarque M Meneghini M. Pretransplant donor-specific IFNgamma ELISPOT as a predictor of graft rejection: A diagnostic test accuracy meta-analysis. Transplant Direct. (2019) 5:e451. doi: 10.1097/TXD.0000000000000886 31165086 PMC6511445

[139] Mendoza Rojas A Verhoeven J de Kuiper R Clahsen-van Groningen MC Boer K Hesselink DA. Alloreactive T cells to assess acute rejection risk in kidney transplant recipients. Transplant Direct. (2023) 9:e1478. doi: 10.1097/TXD.0000000000001478 37096150 PMC10121441

[140] Wojciechowski D Wiseman A. Long-term immunosuppression management: opportunities and uncertainties. Clin J Am Soc Nephrol. (2021) 16:1264–71. doi: 10.2215/CJN.15040920 PMC8455033 33853841

[141] Song T Yin S Jiang Y Huang Z Liu J Wang Z. Increasing time in therapeutic range of tacrolimus in the first year predicts better outcomes in living-donor kidney transplantation. Front Immunol. (2019) 10:2912. doi: 10.3389/fimmu.2019.02912 31921171 PMC6933438

[142] Borni-Duval C Caillard S Olagne J Perrin P Braun-Parvez L Heibel F. Risk factors for BK virus infection in the era of therapeutic drug monitoring. Transplantation. (2013) 95:1498–505. doi: 10.1097/TP.0b013e3182921995 23778568

[143] Davis S Gralla J Klem P Stites E Wiseman A Cooper JE. Tacrolimus intrapatient variability, time in therapeutic range, and risk of *de novo* donor-specific antibodies. Transplantation. (2020) 104:881–7. doi: 10.1097/TP.0000000000002913 32224815

[144] Yin S Song T Jiang Y Li X Fan Y Lin T. Tacrolimus trough level at the first month may predict renal transplantation outcomes among living chinese kidney transplant patients: A propensity score-matched analysis. Ther Drug Monit. (2019) 41:308–16. doi: 10.1097/FTD.0000000000000593 PMC6553958 31083041

[145] Friebus-Kardash J Nela E Mohlendick B Kribben A Siffert W Heinemann FM. Development of *de novo* donor-specific HLA antibodies and AMR in renal transplant patients depends on CYP3A5 genotype. Transplantation. (2022) 106:1031–42. doi: 10.1097/TP.0000000000003871 PMC9038248 34241984

[146] Metz DK Holford N Kausman JY Walker A Cranswick N Staatz CE. Optimizing mycophenolic acid exposure in kidney transplant recipients: time for target concentration intervention. Transplantation. (2019) 103:2012–30. doi: 10.1097/TP.0000000000002762 PMC6756255 31584924

[147] Fu L Huang Z Song T He S Zeng D Rao Z. Short-term therapeutic drug monitoring of mycophenolic acid reduces infection: a prospective, single-center cohort study in Chinese living-related kidney transplantation. Transpl Infect Dis. (2014) 16:760–6. doi: 10.1111/tid.2014.16.issue-5 25092411

[148] Barraclough KA Staatz CE Johnson DW Lee KJ McWhinney BC Ungerer JP. Kidney transplant outcomes are related to tacrolimus, mycophenolic acid and prednisolone exposure in the first week. Transpl Int. (2012) 25:1182–93. doi: 10.1111/j.1432-2277.2012.01553.x 22946513

[149] van Gelder T Tedesco Silva H de Fijter JW Budde K Kuypers D Arns W. Renal transplant patients at high risk of acute rejection benefit from adequate exposure to mycophenolic acid. Transplantation. (2010) 89:595–9. doi: 10.1097/TP.0b013e3181ca7d84 20124953

[150] Langone A Doria C Greenstein S Narayanan M Ueda K Sankari B. Does reduction in mycophenolic acid dose compromise efficacy regardless of tacrolimus exposure level? An analysis of prospective data from the Mycophenolic Renal Transplant (MORE) Registry. Clin Transplant. (2013) 27:15–24. doi: 10.1111/j.1399-0012.2012.01694.x 22861144 PMC3593178

[151] Roufosse C Simmonds N Clahsen-van Groningen M Haas M Henriksen KJ Horsfield C. A 2018 reference guide to the banff classification of renal allograft pathology. Transplantation. (2018) 102:1795–814. doi: 10.1097/TP.0000000000002366 PMC7597974 30028786

[152] Wiebe C Gibson IW Blydt-Hansen TD Pochinco D Birk PE Ho J. Rates and determinants of progression to graft failure in kidney allograft recipients with *de novo* donor-specific antibody. Am J Transplant. (2015) 15:2921–30. doi: 10.1111/ajt.13347 26096305

[153] Wiebe C Pochinco D Blydt-Hansen TD Ho J Birk PE Karpinski M. Class II HLA epitope matching-A strategy to minimize *de novo* donor-specific antibody development and improve outcomes. Am J Transplant. (2013) 13:3114–22. doi: 10.1111/ajt.12478 24164958

[154] Zhang R. Donor-specific antibodies in kidney transplant recipients. Clin J Am Soc Nephrol. (2018) 13:182–92. doi: 10.2215/CJN.00700117 PMC5753302 28446536

[155] Schinstock CA Mannon RB Budde K Chong AS Haas M Knechtle S. Recommended treatment for antibody-mediated rejection after kidney transplantation: the 2019 expert consensus from the transplantion society working group. Transplantation. (2020) 104:911–22. doi: 10.1097/TP.0000000000003095 PMC7176344 31895348

[156] Tambur AR Herrera ND Haarberg KM Cusick MF Gordon RA Leventhal JR. Assessing antibody strength: comparison of MFI, C1q, and titer information. Am J Transplant. (2015) 15:2421–30. doi: 10.1111/ajt.13295 25930984

[157] Kardol-Hoefnagel T Otten HG. A comprehensive overview of the clinical relevance and treatment options for antibody-mediated rejection associated with non-HLA antibodies. Transplantation. (2021) 105:1459–70. doi: 10.1097/TP.0000000000003551 PMC8221725 33208690

[158] Hoshino J Kaneku H Everly MJ Greenland S Terasaki PI. Using donor-specific antibodies to monitor the need for immunosuppression. Transplantation. (2012) 93:1173–8. doi: 10.1097/TP.0b013e31824f3d7c 22592887

[159] Sellares J de Freitas DG Mengel M Reeve J Einecke G Sis B. Understanding the causes of kidney transplant failure: the dominant role of antibody-mediated rejection and nonadherence. Am J Transplant. (2012) 12:388–99. doi: 10.1111/j.1600-6143.2011.03840.x 22081892

[160] Guidicelli G Guerville F Lepreux S Wiebe C Thaunat O Dubois V. Non-complement-binding *de novo* donor-specific anti-HLA antibodies and kidney allograft survival. J Am Soc Nephrol. (2016) 27:615–25. doi: 10.1681/ASN.2014040326 PMC4731103 26047793

[161] Ntokou IS Iniotaki AG Kontou EN Darema MN Apostolaki MD Kostakis AG. Long-term follow up for anti-HLA donor specific antibodies postrenal transplantation: high immunogenicity of HLA class II graft molecules. Transpl Int. (2011) 24:1084–93. doi: 10.1111/j.1432-2277.2011.01312.x 21848902

[162] Wiebe C Gibson IW Blydt-Hansen TD Karpinski M Ho J Storsley LJ. Evolution and clinical pathologic correlations of *de novo* donor-specific HLA antibody post kidney transplant. Am J Transplant. (2012) 12:1157–67. doi: 10.1111/j.1600-6143.2012.04013.x 22429309

[163] Taber DJ Su Z Fleming JN McGillicuddy JW Posadas-Salas MA Treiber FA. Tacrolimus trough concentration variability and disparities in african american kidney transplantation. Transplantation. (2017) 101:2931–8. doi: 10.1097/TP.0000000000001840 PMC5709143 28658199

[164] Wiebe C Rush DN Nevins TE Birk PE Blydt-Hansen T Gibson IW. Class II eplet mismatch modulates tacrolimus trough levels required to prevent donor-specific antibody development. J Am Soc Nephrol. (2017) 28:3353–62. doi: 10.1681/ASN.2017030287 PMC5661295 28729289

[165] Halloran PF Madill-Thomsen KS Pon S Sikosana MLN Bohmig GA Bromberg J. Molecular diagnosis of ABMR with or without donor-specific antibody in kidney transplant biopsies: Differences in timing and intensity but similar mechanisms and outcomes. Am J Transplant. (2022) 22:1976–91. doi: 10.1111/ajt.17092 PMC9540308 35575435

[166] Bloom RD Bromberg JS Poggio ED Bunnapradist S Langone AJ Sood P. Cell-free DNA and active rejection in kidney allografts. J Am Soc Nephrol. (2017) 28:2221–32. doi: 10.1681/ASN.2016091034 PMC5491290 28280140

[167] Garcia Moreira V Prieto Garcia B Baltar Martin JM Ortega Suarez F Alvarez FV. Cell-free DNA as a noninvasive acute rejection marker in renal transplantation. Clin Chem. (2009) 55:1958–66. doi: 10.1373/clinchem.2009.129072 19729469

[168] Oellerich M Budde K Osmanodja B Bornemann-Kolatzki K Beck J Schutz E. Donor-derived cell-free DNA as a diagnostic tool in transplantation. Front Genet. (2022) 13:1031894. doi: 10.3389/fgene.2022.1031894 36339004 PMC9634115

[169] Oellerich M Sherwood K Keown P Schutz E Beck J Stegbauer J. Liquid biopsies: donor-derived cell-free DNA for the detection of kidney allograft injury. Nat Rev Nephrol. (2021) 17:591–603. doi: 10.1038/s41581-021-00428-0 34031575

[170] Aubert O Ursule-Dufait C Brousse R Gueguen J Racape M Raynaud M. Cell-free DNA for the detection of kidney allograft rejection. Nat Med. (2024) 30:2320–7. doi: 10.1038/s41591-024-03087-3 PMC11333280 38824959

[171] Eikmans M Gielis EM Ledeganck KJ Yang J Abramowicz D Claas FFJ. Non-invasive biomarkers of acute rejection in kidney transplantation: novel targets and strategies. Front Med (Lausanne). (2018) 5:358. doi: 10.3389/fmed.2018.00358 30671435 PMC6331461

[172] Sigdel TK Vitalone MJ Tran TQ Dai H Hsieh SC Salvatierra O. A rapid noninvasive assay for the detection of renal transplant injury. Transplantation. (2013) 96:97–101. doi: 10.1097/TP.0b013e318295ee5a 23756769 PMC4472435

[173] Goussous N Xie W Dawany N Scalea JR Bartosic A Haririan A. Donor-derived cell-free DNA in infections in kidney transplant recipients: case series. Transplant Direct. (2020) 6:e568. doi: 10.1097/TXD.0000000000001019 32766423 PMC7339327

[174] Bazemore K Permpalung N Mathew J Lemma M Haile B Avery R. Elevated cell-free DNA in respiratory viral infection and associated lung allograft dysfunction. Am J Transplant. (2022) 22:2560–70. doi: 10.1111/ajt.17125 35729715

[175] Alam AH Van Zyl J Shakoor HI Farsakh D Abdelrehim AB Maliakkal N. The impact of active cytomegalovirus infection on donor-derived cell-free DNA testing in heart transplant recipients. Clin Transplant. (2024) 38:e15287. doi: 10.1111/ctr.15287 38477177

[176] Sperry BW Khumri TM Kao AC. Donor-derived cell-free DNA in a heart transplant patient with COVID-19. Clin Transplant. (2020) 34:e14070. doi: 10.1111/ctr.v34.11 32856335 PMC7460935

[177] Levitsky J Kandpal M Guo K Kleiboeker S Sinha R Abecassis M. Donor-derived cell-free DNA levels predict graft injury in liver transplant recipients. Am J Transplant. (2022) 22:532–40. doi: 10.1111/ajt.16835 34510731

[178] Jana K Rammohan A Ramani A Gunasekaran B Vij M Ramamoorthi M. Role of donor-derived cell-free DNA in predicting short-term allograft health in liver transplant recipients. J Clin Exp Hepatol. (2024) 14:101477. doi: 10.1016/j.jceh.2024.101477 39170833 PMC11334858

[179] Li Y Liang B. Circulating donor-derived cell-free DNA as a marker for rejection after lung transplantation. Front Immunol. (2023) 14:1263389. doi: 10.3389/fimmu.2023.1263389 37885888 PMC10598712

[180] Keller MB Newman D Alnababteh M Ponor L Shah P Mathew J. Extreme elevations of donor-derived cell-free DNA increases the risk of chronic lung allograft dysfunction and death, even without clinical manifestations of disease. J Heart Lung Transplant. (2024) 43:1374–82. doi: 10.1016/j.healun.2024.04.064 PMC13186095 38705500

[181] Keller M Sun J Mutebi C Shah P Levine D Aryal S. Donor-derived cell-free DNA as a composite marker of acute lung allograft dysfunction in clinical care. J Heart Lung Transplant. (2022) 41:458–66. doi: 10.1016/j.healun.2021.12.009 35063338

[182] Kim PJ Olymbios M Siu A Wever Pinzon O Adler E Liang N. A novel donor-derived cell-free DNA assay for the detection of acute rejection in heart transplantation. J Heart Lung Transplant. (2022) 41:919–27. doi: 10.1016/j.healun.2022.04.002 PMC9670834 35577713

[183] Khush KK Patel J Pinney S Kao A Alharethi R DePasquale E. Noninvasive detection of graft injury after heart transplant using donor-derived cell-free DNA: A prospective multicenter study. Am J Transplant. (2019) 19:2889–99. doi: 10.1111/ajt.15339 PMC6790566 30835940

[184] Xing Y Guo Q Wang C Shi H Zheng J Jia Y. Donor-derived cell-free DNA as a diagnostic marker for kidney-allograft rejection: A systematic review and meta-analysis. Biomol Biomed. (2024) 24:731–40. doi: 10.17305/bb.2024.10049 PMC11293223 38386614

[185] Park S Sellares J Tinel C Anglicheau D Bestard O Friedewald JJ. European society of organ transplantation consensus statement on testing for non-invasive diagnosis of kidney allograft rejection. Transpl Int. (2023) 36:12115. doi: 10.3389/ti.2023.12115 38239762 PMC10794444

[186] Stites E Kumar D Olaitan O John Swanson S Leca N Weir M. High levels of dd-cfDNA identify patients with TCMR 1A and borderline allograft rejection at elevated risk of graft injury. Am J Transplant. (2020) 20:2491–8. doi: 10.1111/ajt.15822 PMC7496411 32056331

[187] Huang E Mengel M Clahsen-van Groningen MC Jackson AM. Diagnostic potential of minimally invasive biomarkers: A biopsy-centered viewpoint from the banff minimally invasive diagnostics working group. Transplantation. (2023) 107:45–52. doi: 10.1097/TP.0000000000004339 36508645 PMC9746335

[188] Halloran PF Reeve J Madill-Thomsen KS Demko Z Prewett A Gauthier P. Antibody-mediated rejection without detectable donor-specific antibody releases donor-derived cell-free DNA: results from the trifecta study. Transplantation. (2023) 107:709–19. doi: 10.1097/TP.0000000000004324 PMC9946174 36190186

[189] Xiao H Gao F Pang Q Xia Q Zeng X Peng J. Diagnostic accuracy of donor-derived cell-free DNA in renal-allograft rejection: A meta-analysis. Transplantation. (2021) 105:1303–10. doi: 10.1097/TP.0000000000003443 32890130

[190] Shen J Zhou Y Chen Y Li X Lei W Ge J. Dynamics of early post-operative plasma ddcfDNA levels in kidney transplantation: a single-center pilot study. Transpl Int. (2019) 32:184–92. doi: 10.1111/tri.2019.32.issue-2 30198148

[191] Graver AS Lee D Power DA Whitlam JB. Understanding donor-derived cell-free DNA in kidney transplantation: an overview and case-based guide for clinicians. Transplantation. (2023) 107:1675–86. doi: 10.1097/TP.0000000000004482 36579675

[192] Sherwood K Weimer ET. Characteristics, properties, and potential applications of circulating cell-free dna in clinical diagnostics: a focus on transplantation. J Immunol Methods. (2018) 463:27–38. doi: 10.1016/j.jim.2018.09.011 30267663

[193] Sun K Jiang P Chan KC Wong J Cheng YK Liang RH. Plasma DNA tissue mapping by genome-wide methylation sequencing for noninvasive prenatal, cancer, and transplantation assessments. Proc Natl Acad Sci U S A. (2015) 112:E5503–12. doi: 10.1073/pnas.1508736112 PMC4603482 26392541

[194] Paul RS Almokayad I Collins A Raj D Jagadeesan M. Donor-derived cell-free DNA: advancing a novel assay to new heights in renal transplantation. Transplant Direct. (2021) 7:e664. doi: 10.1097/TXD.0000000000001098 33564715 PMC7862009

[195] Hricik DE Nickerson P Formica RN Poggio ED Rush D Newell KA. Multicenter validation of urinary CXCL9 as a risk-stratifying biomarker for kidney transplant injury. Am J Transplant. (2013) 13:2634–44. doi: 10.1111/ajt.12426 PMC3959786 23968332

[196] Rabant M Amrouche L Lebreton X Aulagnon F Benon A Sauvaget V. Urinary C-X-C motif chemokine 10 independently improves the noninvasive diagnosis of antibody-mediated kidney allograft rejection. J Am Soc Nephrol. (2015) 26:2840–51. doi: 10.1681/ASN.2014080797 PMC4625672 25948873

[197] Jackson JA Kim EJ Begley B Cheeseman J Harden T Perez SD. Urinary chemokines CXCL9 and CXCL10 are noninvasive markers of renal allograft rejection and BK viral infection. Am J Transplant. (2011) 11:2228–34. doi: 10.1111/j.1600-6143.2011.03680.x PMC3184377 21812928

[198] Tinel C Devresse A Vermorel A Sauvaget V Marx D Avettand-Fenoel V. Development and validation of an optimized integrative model using urinary chemokines for noninvasive diagnosis of acute allograft rejection. Am J Transplant. (2020) 20:3462–76. doi: 10.1111/ajt.15959 32342614

[199] Tinel C Vermorel A Picciotto D Morin L Devresse A Sauvaget V. Deciphering the prognostic and predictive value of urinary CXCL10 in kidney recipients with BK virus reactivation. Front Immunol. (2020) 11:604353. doi: 10.3389/fimmu.2020.604353 33362789 PMC7759001

[200] Ho J Schaub S Wiebe C Gao A Wehmeier C Koller MT. Urinary CXCL10 chemokine is associated with alloimmune and virus compartment-specific renal allograft inflammation. Transplantation. (2018) 102:521–9. doi: 10.1097/TP.0000000000001931 28902772

[201] Rabant M Amrouche L Morin L Bonifay R Lebreton X Aouni L. Early low urinary CXCL9 and CXCL10 might predict immunological quiescence in clinically and histologically stable kidney recipients. Am J Transplant. (2016) 16:1868–81. doi: 10.1111/ajt.13677 26694099

[202] Blydt-Hansen TD Sharma A Gibson IW Wiebe C Sharma AP Langlois V. Validity and utility of urinary CXCL10/Cr immune monitoring in pediatric kidney transplant recipients. Am J Transplant. (2021) 21:1545–55. doi: 10.1111/ajt.16336 33034126

[203] Shino MY Todd JL Neely ML Kirchner J Frankel CW Snyder LD. Plasma CXCL9 and CXCL10 at allograft injury predict chronic lung allograft dysfunction. Am J Transplant. (2022) 22:2169–79. doi: 10.1111/ajt.17108 PMC9427677 35634722

[204] Friedman BH Wolf JH Wang L Putt ME Shaked A Christie JD. Serum cytokine profiles associated with early allograft dysfunction in patients undergoing liver transplantation. Liver Transpl. (2012) 18:166–76. doi: 10.1002/lt.22451 PMC3266982 22006860

[205] Rosenblum JM Zhang QW Siu G Collins TL Sullivan T Dairaghi DJ. CXCR3 antagonism impairs the development of donor-reactive, IFN-gamma-producing effectors and prolongs allograft survival. Transplantation. (2009) 87:360–9. doi: 10.1097/TP.0b013e31819574e9 PMC2738925 19202440

[206] Hirt-Minkowski P Handschin J Stampf S Hopfer H Menter T Senn L. Randomized trial to assess the clinical utility of renal allograft monitoring by urine CXCL10 chemokine. J Am Soc Nephrol. (2023) 34:1456–69. doi: 10.1681/ASN.0000000000000160 PMC10400101 37228005

[207] Adam BA Kikic Z Wagner S Bouatou Y Gueguen J Drieux F. Intragraft gene expression in native kidney BK virus nephropathy versus T cell-mediated rejection: Prospects for molecular diagnosis and risk prediction. Am J Transplant. (2020) 20:3486–501. doi: 10.1111/ajt.15980 32372431

[208] Ross MH Zick BL Tsalik EL. Host-based diagnostics for acute respiratory infections. Clin Ther. (2019) 41:1923–38. doi: 10.1016/j.clinthera.2019.06.007 31353133

[209] Steinbrink JM LY Gray A Mohamedaly O Zick B McClain MT. Host gene expression biomarkers to distinguish between causes of acute respiratory symptoms in lung transplant recipients. Open Forum Infect Dis. (2021) 4. doi: 10.1093/ofid/ofab466.1217

[210] Halloran PF Madill-Thomsen KS Reeve J. The molecular phenotype of kidney transplants: insights from the MMDx project. Transplantation. (2024) 108:45–71. doi: 10.1097/TP.0000000000004624 37310258 PMC10718223

[211] Madill-Thomsen K Perkowska-Ptasinska A Bohmig GA Eskandary F Einecke G Gupta G. Discrepancy analysis comparing molecular and histology diagnoses in kidney transplant biopsies. Am J Transplant. (2020) 20:1341–50. doi: 10.1111/ajt.15752 31846554

[212] Hidalgo LG Sis B Sellares J Campbell PM Mengel M Einecke G. NK cell transcripts and NK cells in kidney biopsies from patients with donor-specific antibodies: evidence for NK cell involvement in antibody-mediated rejection. Am J Transplant. (2010) 10:1812–22. doi: 10.1111/j.1600-6143.2010.03201.x 20659089

[213] Halloran PF Reeve JP Pereira AB Hidalgo LG Famulski KS. Antibody-mediated rejection, T cell-mediated rejection, and the injury-repair response: new insights from the Genome Canada studies of kidney transplant biopsies. Kidney Int. (2014) 85:258–64. doi: 10.1038/ki.2013.300 23965521

[214] Mengel M Loupy A Haas M Roufosse C Naesens M Akalin E. Banff 2019 Meeting Report: Molecular diagnostics in solid organ transplantation-Consensus for the Banff Human Organ Transplant (B-HOT) gene panel and open source multicenter validation. Am J Transplant. (2020) 20:2305–17. doi: 10.1111/ajt.16059 PMC7496585 32428337

[215] Deng MC Eisen HJ Mehra MR Billingham M Marboe CC Berry G. Noninvasive discrimination of rejection in cardiac allograft recipients using gene expression profiling. Am J Transplant. (2006) 6:150–60. doi: 10.1111/j.1600-6143.2005.01175.x 16433769

[216] Kobashigawa J Patel J Azarbal B Kittleson M Chang D Czer L. Randomized pilot trial of gene expression profiling versus heart biopsy in the first year after heart transplant: early invasive monitoring attenuation through gene expression trial. Circ Heart Fail. (2015) 8:557–64. doi: 10.1161/CIRCHEARTFAILURE.114.001658 25697852

[217] Pham MX Teuteberg JJ Kfoury AG Starling RC Deng MC Cappola TP. Gene-expression profiling for rejection surveillance after cardiac transplantation. N Engl J Med. (2010) 362:1890–900. doi: 10.1056/NEJMoa0912965 20413602

[218] Moayedi Y Foroutan F Miller RJH Fan CS Posada JGD Alhussein M. Risk evaluation using gene expression screening to monitor for acute cellular rejection in heart transplant recipients. J Heart Lung Transplant. (2019) 38:51–8. doi: 10.1016/j.healun.2018.09.004 30352779

[219] Akalin E Weir MR Bunnapradist S Brennan DC Delos Santos R Langone A. Clinical validation of an immune quiescence gene expression signature in kidney transplantation. Kidney360. (2021) 2:1998–2009. doi: 10.34067/KID.0005062021 35419538 PMC8986041

[220] Roedder S Sigdel T Salomonis N Hsieh S Dai H Bestard O. The kSORT assay to detect renal transplant patients at high risk for acute rejection: results of the multicenter AART study. PloS Med. (2014) 11:e1001759. doi: 10.1371/journal.pmed.1001759 25386950 PMC4227654

[221] Van Loon E Giral M Anglicheau D Lerut E Dubois V Rabeyrin M. Diagnostic performance of kSORT, a blood-based mRNA assay for noninvasive detection of rejection after kidney transplantation: A retrospective multicenter cohort study. Am J Transplant. (2021) 21:740–50. doi: 10.1111/ajt.16179 32627407

[222] Friedewald JJ Kurian SM Heilman RL Whisenant TC Poggio ED Marsh C. Development and clinical validity of a novel blood-based molecular biomarker for subclinical acute rejection following kidney transplant. Am J Transplant. (2019) 19:98–109. doi: 10.1111/ajt.15011 29985559 PMC6387870

[223] Khatri P Roedder S Kimura N De Vusser K Morgan AA Gong Y. A common rejection module (CRM) for acute rejection across multiple organs identifies novel therapeutics for organ transplantation. J Exp Med. (2013) 210:2205–21. doi: 10.1084/jem.20122709 PMC3804941 24127489

[224] Sigdel TK Yang JYC Bestard O Schroeder A Hsieh SC Liberto JM. A urinary Common Rejection Module (uCRM) score for non-invasive kidney transplant monitoring. PloS One. (2019) 14:2:e022005. doi: 10.1371/journal.pone.0220052 PMC6668802 31365568

[225] Zarinsefat A Guerra JMA Sigdel T Damm I Sarwal R Chan-On C. Use of the tissue common rejection module score in kidney transplant as an objective measure of allograft inflammation. Front Immunol. (2020) 11:614343. doi: 10.3389/fimmu.2020.614343 33613539 PMC7886808

[226] Hamdorf M Kawakita S Everly M. The potential of microRNAs as novel biomarkers for transplant rejection. J Immunol Res. (2017) 2017:4072364. doi: 10.1155/2017/4072364 28191475 PMC5278203

[227] Mas VR Dumur CI Scian MJ Gehrau RC Maluf DG. MicroRNAs as biomarkers in solid organ transplantation. Am J Transplant. (2013) 13:11–9. doi: 10.1111/j.1600-6143.2012.04313.x PMC3927320 23136949

[228] Chancharoenthana W Traitanon O Leelahavanichkul A Tasanarong A. Molecular immune monitoring in kidney transplant rejection: a state-of-the-art review. Front Immunol. (2023) 14:1206929. doi: 10.3389/fimmu.2023.1206929 37675106 PMC10477600

[229] Ambros V Bartel B Bartel DP Burge CB Carrington JC Chen X. A uniform system for microRNA annotation. RNA. (2003) 9:277–9. doi: 10.1261/rna.2183803 PMC1370393 12592000

[230] Virtanen E Seppala H Helantera I Laine P Lautenschlager I Paulin L. BK polyomavirus microRNA expression and sequence variation in polyomavirus-associated nephropathy. J Clin Virol. (2018) 102:70–6. doi: 10.1016/j.jcv.2018.02.007 29518695

[231] Demey B Descamps V Presne C Helle F Francois C Duverlie G. BK polyomavirus micro-RNAs: time course and clinical relevance in kidney transplant recipients. Viruses. (2021) 13:351. doi: 10.3390/v13020351 33672313 PMC7926448

[232] Demey B Bentz M Descamps V Morel V Francois C Castelain S. BK Polyomavirus bkv-miR-B1-5p: A Stable Micro-RNA to Monitor Active Viral Replication after Kidney Transplantation. Int J Mol Sci. (2022) 23:7240. doi: 10.3390/ijms23137240 35806242 PMC9266457

[233] Bauman Y Nachmani D Vitenshtein A Tsukerman P Drayman N Stern-Ginossar N. An identical miRNA of the human JC and BK polyoma viruses targets the stress-induced ligand ULBP3 to escape immune elimination. Cell Host Microbe. (2011) 9:93–102. doi: 10.1016/j.chom.2011.01.008 21320692

[234] Afshari A Yaghobi R Golshan M. Cytomegalovirus microRNAs level determination in kidney recipients post transplantation. Virol J. (2022) 19:147. doi: 10.1186/s12985-022-01880-5 36096838 PMC9465962

[235] Fernandez-Ruiz M Lopez-Garcia A Valverde-Manso A Parra P Rodriguez-Goncer I Ruiz-Merlo T. Human microRNA sequencing and cytomegalovirus infection risk after kidney transplantation. Am J Transplant. (2024) 24:1180–92. doi: 10.1016/j.ajt.2024.01.028 38311311

[236] Palleschi A Gaudioso G Edefonti V Musso V Terrasi A Ambrogi F. Bronchoalveolar lavage-microRNAs are potential novel biomarkers of outcome after lung transplantation. Transplant Direct. (2020) 6:e547. doi: 10.1097/TXD.0000000000000994 32548241 PMC7213607

[237] Gohir W Klement W Singer LG Palmer SM Mazzulli T Keshavjee S. Identifying host microRNAs in bronchoalveolar lavage samples from lung transplant recipients infected with Aspergillus. J Heart Lung Transplant. (2020) 39:1228–37. doi: 10.1016/j.healun.2020.07.014 PMC9453783 32771440

[238] Oghumu S Bracewell A Nori U Maclean KH Balada-Lasat JM Brodsky S. Acute pyelonephritis in renal allografts: a new role for microRNAs? Transplantation. (2014) 97:559–68. doi: 10.1097/01.TP.0000441322.95539.b3 PMC4090250 24521778

[239] Wei L Wang M Qu X Mah A Xiong X Harris AG. Differential expression of microRNAs during allograft rejection. Am J Transplant. (2012) 12:1113–23. doi: 10.1111/j.1600-6143.2011.03958.x PMC3461331 22300508

[240] Farid WR Verhoeven CJ de Jonge J Metselaar HJ Kazemier G van der Laan LJ. The ins and outs of microRNAs as biomarkers in liver disease and transplantation. Transpl Int. (2014) 27:1222–32. doi: 10.1111/tri.12379 24963540

[241] Shaked A Chang BL Barnes MR Sayre P Li YR Asare S. An ectopically expressed serum miRNA signature is prognostic, diagnostic, and biologically related to liver allograft rejection. Hepatology. (2017) 65:269–80. doi: 10.1002/hep.28786 27533743

[242] Millan O Ruiz P Orts L Ferre P Crespo G Santana M. Monitoring of miR-181a-5p and miR-155-5p Plasmatic Expression as Prognostic Biomarkers for Acute and Subclinical Rejection in *de novo* Adult Liver Transplant Recipients. Front Immunol. (2019) 10:873. doi: 10.3389/fimmu.2019.00873 31068943 PMC6491707

[243] Shah P Agbor-Enoh S Bagchi P deFilippi CR Mercado A Diao G. Circulating microRNAs in cellular and antibody-mediated heart transplant rejection. J Heart Lung Transplant. (2022) 41:1401–13. doi: 10.1016/j.healun.2022.06.019 PMC9529890 35872109

[244] Constanso-Conde I Hermida-Prieto M Barge-Caballero E Nunez L Pombo-Otero J Suarez-Fuentetaja N. Circulating miR-181a-5p as a new biomarker for acute cellular rejection in heart transplantation. J Heart Lung Transplant. (2020) 39:1100–8. doi: 10.1016/j.healun.2020.05.018 32654912

[245] Kennel PJ Yahi A Naka Y Mancini DM Marboe CC Max K. Longitudinal profiling of circulating miRNA during cardiac allograft rejection: a proof-of-concept study. ESC Heart Fail. (2021) 8:1840–9. doi: 10.1002/ehf2.13238 PMC8120386 33713567

[246] Coutance G Racape M Baudry G Lecuyer L Roubille F Blanchart K. Validation of the clinical utility of microRNA as noninvasive biomarkers of cardiac allograft rejection: A prospective longitudinal multicenter study. J Heart Lung Transplant. (2023) 42:1505–9. doi: 10.1016/j.healun.2023.07.010 37487804

[247] Miyahara N Benazzo A Oberndorfer F Iwasaki A Laszlo V Dome B. MiR-21 in lung transplant recipients with chronic lung allograft dysfunction. Transpl Int. (2021) 35:10184. doi: 10.3389/ti.2021.10184 35185369 PMC8842266

[248] Ladak SS Ward C Ali S. The potential role of microRNAs in lung allograft rejection. J Heart Lung Transplant. (2016) 35:550–9. doi: 10.1016/j.healun.2016.03.018 27197771

[249] Xu Z Nayak D Yang W Baskaran G Ramachandran S Sarma N. Dysregulated microRNA expression and chronic lung allograft rejection in recipients with antibodies to donor HLA. Am J Transplant. (2015) 15:1933–47. doi: 10.1111/ajt.13185 PMC5607954 25649290

[250] Millan O Budde K Sommerer C Aliart I Rissling O Bardaji B. Urinary miR-155-5p and CXCL10 as prognostic and predictive biomarkers of rejection, graft outcome and treatment response in kidney transplantation. Br J Clin Pharmacol. (2017) 83:2636–50. doi: 10.1111/bcp.13399 PMC5698579 28880456

[251] Nagy PF Pocsi M Fejes Z Bidiga L Szabo E Balogh O. Investigation of circulating microRNA levels in antibody-mediated rejection after kidney transplantation. Transplant Proc. (2022) 54:2570–7. doi: 10.1016/j.transproceed.2022.10.044 36400592

[252] Soltaninejad E Nicknam MH Nafar M Ahmadpoor P Pourrezagholi F Sharbafi MH. Differential expression of microRNAs in renal transplant patients with acute T-cell mediated rejection. Transpl Immunol. (2015) 33:1–6. doi: 10.1016/j.trim.2015.05.002 26002284

[253] Benichou G Wang M Ahrens K Madsen JC. Extracellular vesicles in allograft rejection and tolerance. Cell Immunol. (2020) 349:104063. doi: 10.1016/j.cellimm.2020.104063 32087929 PMC7231511

[254] Gonzalez-Nolasco B Wang M Prunevieille A Benichou G. Emerging role of exosomes in allorecognition and allograft rejection. Curr Opin Organ Transplant. (2018) 23:22–7. doi: 10.1097/MOT.0000000000000489 PMC5972078 29189413

[255] El Fekih R Hurley J Tadigotla V Alghamdi A Srivastava A Coticchia C. Discovery and validation of a urinary exosome mRNA signature for the diagnosis of human kidney transplant rejection. J Am Soc Nephrol. (2021) 32:994–1004. doi: 10.1681/ASN.2020060850 33658284 PMC8017553

[256] Sigdel TK Ng YW Lee S Nicora CD Qian WJ Smith RD. Perturbations in the urinary exosome in transplant rejection. Front Med (Lausanne). (2014) 1:57. doi: 10.3389/fmed.2014.00057 25593928 PMC4292055

[257] Tower CM Reyes M Nelson K Leca N Kieran N Muczynski K. Plasma C4d+ Endothelial microvesicles increase in acute antibody-mediated rejection. Transplantation. (2017) 101:2235–43. doi: 10.1097/TP.0000000000001572 27846156

[258] Kennel PJ Saha A Maldonado DA Givens R Brunjes DL Castillero E. Serum exosomal protein profiling for the non-invasive detection of cardiac allograft rejection. J Heart Lung Transplant. (2018) 37:409–17. doi: 10.1016/j.healun.2017.07.012 28789823

[259] Sukma Dewi I Celik S Karlsson A Hollander Z Lam K McManus JW. Exosomal miR-142-3p is increased during cardiac allograft rejection and augments vascular permeability through down-regulation of endothelial RAB11FIP2 expression. Cardiovasc Res. (2017) 113:440–52. doi: 10.1093/cvr/cvw244 28073833

[260] Sharma M Gunasekaran M Ravichandran R Fisher CE Limaye AP Hu C. Circulating exosomes with lung self-antigens as a biomarker for chronic lung allograft dysfunction: A retrospective analysis. J Heart Lung Transplant. (2020) 39:1210–9. doi: 10.1016/j.healun.2020.07.001 PMC7790863 32713614

[261] Gunasekaran M Xu Z Nayak DK Sharma M Hachem R Walia R. Donor-derived exosomes with lung self-antigens in human lung allograft rejection. Am J Transplant. (2017) 17:474–84. doi: 10.1111/ajt.13915 PMC5340154 27278097

[262] Park S Guo K Heilman RL Poggio ED Taber DJ Marsh CL. Combining blood gene expression and cellfree DNA to diagnose subclinical rejection in kidney transplant recipients. Clin J Am Soc Nephrol. (2021) 16:1539–51. doi: 10.2215/CJN.05530421 PMC8499014 34620649

[263] Henricksen EJ Moayedi Y Purewal S Twiggs JV Waddell K Luikart H. Combining donor derived cell free DNA and gene expression profiling for non-invasive surveillance after heart transplantation. Clin Transplant. (2023) 37:e14699. doi: 10.1111/ctr.14699 35559582

[264] Crespo E Roedder S Sigdel T Hsieh SC Luque S Cruzado JM. Molecular and functional noninvasive immune monitoring in the ESCAPE study for prediction of subclinical renal allograft rejection. Transplantation. (2017) 101:1400–9. doi: 10.1097/TP.0000000000001287 27362314

[265] Van Loon E Tinel C de Loor H Bossuyt X Callemeyn J Coemans M. Automated urinary chemokine assays for noninvasive detection of kidney transplant rejection: A prospective cohort study. Am J Kidney Dis. (2024) 83:467–76. doi: 10.1053/j.ajkd.2023.07.022 37777058

[266] Millan O Ruiz P Julian J Lizana A Fundora Y Crespo G. A plasmatic score using a miRNA signature and CXCL-10 for accurate prediction and diagnosis of liver allograft rejection. Front Immunol. (2023) 14:1196882. doi: 10.3389/fimmu.2023.1196882 37325660 PMC10265684

[267] Gielis EM Anholts JDH van Beelen E Haasnoot GW De Fijter HW Bajema I. A combined microRNA and chemokine profile in urine to identify rejection after kidney transplantation. Transplant Direct. (2021) 7:e711. doi: 10.1097/TXD.0000000000001169 34131583 PMC8196093

[268] Hricik DE Augustine J Nickerson P Formica RN Poggio ED Rush D. Interferon gamma ELISPOT testing as a risk-stratifying biomarker for kidney transplant injury: results from the CTOT-01 multicenter study. Am J Transplant. (2015) 15:3166–73. doi: 10.1111/ajt.13401 PMC4946339 26226830

[269] Tambur AR Kosmoliaptsis V Claas FHJ Mannon RB Nickerson P Naesens M. Significance of HLA-DQ in kidney transplantation: time to reevaluate human leukocyte antigen-matching priorities to improve transplant outcomes? An expert review and recommendations. Kidney Int. (2021) 100:1012–22. doi: 10.1016/j.kint.2021.06.026 34246656

[270] Boyarsky BJ Werbel WA Avery RK Tobian AAR Massie AB Segev DL. Antibody response to 2-dose SARS-coV-2 mRNA vaccine series in solid organ transplant recipients. JAMA. (2021) 325:2204–6. doi: 10.1001/jama.2021.7489 PMC8100911 33950155

[271] Chaudhry D Chaudhry A Peracha J Sharif A. Survival for waitlisted kidney failure patients receiving transplantation versus remaining on waiting list: systematic review and meta-analysis. BMJ. (2022) 376:e068769. doi: 10.1136/bmj-2021-068769 35232772 PMC8886447

[272] Norden R Magnusson J Lundin A Tang KW Nilsson S Lindh M. Quantification of torque teno virus and epstein-barr virus is of limited value for predicting the net state of immunosuppression after lung transplantation. Open Forum Infect Dis. (2018) 5:ofy050. doi: 10.1093/ofid/ofy050 29644247 PMC5888719

[273] Steinbrink JM Zaas AK Betancourt M Modliszewski JL Corcoran DL McClain MT. A transcriptional signature accurately identifies Aspergillus Infection across healthy and immunosuppressed states. Transl Res. (2020) 219:1–12. doi: 10.1016/j.trsl.2020.02.005 32165060 PMC7170547

[274] Zhang W Yi Z Keung KL Shang H Wei C Cravedi P. A peripheral blood gene expression signature to diagnose subclinical acute rejection. J Am Soc Nephrol. (2019) 30:1481–94. doi: 10.1681/ASN.2018111098 PMC6683710 31278196

[275] Tinel C Sauvaget V Aouni L Lamarthee B Terzi F Legendre C. Transforming kidney transplant monitoring with urine CXCL9 and CXCL10: practical clinical implementation. Sci Rep. (2024) 14:20357. doi: 10.1038/s41598-024-70390-x 39223175 PMC11369285

