# Supplementary material for: Current and emerging tools for simultaneous assessment of infection and rejection risk in transplantation
Source: Front Immunol. 2024 Nov 26;15:1490472. doi: 10.3389/fimmu.2024.1490472 (PMC11628869; doi:10.3389/fimmu.2024.1490472)
Supplement: Supplementary file 1 [file Table1.pdf]

Supplementary table 1. Studies on immune markers and their association with **infection (blue)** and **rejection (red).**

| Immune marker | Organ (participant number) | Study design | Results |
|---|---|---|---|
| **Absolute Lymphocyte count (ALC)** | Liver transplant (449) | Retrospective cohort | Pre-transplant lymphopenia associated with higher short-term mortality and longer-term infection risk(77). |
| | Heart transplant (375) | Retrospective cohort | ALC (≤0.75 x $10^3$ cells/µL) at 1-month strongly correlates with higher risk of infections and death in the subsequent 11 months(78). |
| | Heart transplant (158) | Retrospective cohort | 50% decrease in ALC and/or ALC ≤610 cells/µL associated with increased risk of CMV(79). |
| | Kidney & Kidney/Pancreas (2999) | Prospective cohort | ALC <750/$mm^3$ 1-year post-transplant associated with increased viral infection, death and graft failure(80). |
| | Heart/Liver/Kidney (170) | Retrospective cohort | Lymphopenia at the end of CMV treatment strongly predicted risk of CMV recurrence. (unadjusted HR-1.1 for every 100cell/µL drop in ALC)(58). |
| **CD4+** | Lung transplant (83) | Prospective cohort | CD4 T-cell count nadir <200 cells/µl 3 months post-transplant associated with increased risk of viral infections(81). |
| | Kidney (100) | Prospective cross-sectional | Higher CD4 count (>497 CD4+ cells/µL) increased risk of rejection(82). |
| **CD4+ and CD8+ CD4+/CD8 ratio** | Heart (48) & Kidney (42) | Retrospective cohort | Heart transplant recipients who developed OI had lower nadir CD4+ (<200 cells/µl) and CD8+ counts. KTRs with OI had lower CD8+ counts(83). |
| | Kidney (304) | Prospective cohort | Low CD8+ and CD4+ counts (<0.100 x $10^3$ cell/µL) post anti-thymocyte globulin therapy associated with increased risk of opportunistic infections and CMV disease(57). |
| | SOT (130) | Retrospective cohort | Impaired CMV specific and non-specific CD8+ function score (quantitative and qualitative evaluation) associated with CMV infection and tissue invasive disease(84). |

| | | | |
|---|---|---|---|
| | Heart transplant (7) | Prospective cohort | An increase in the effector T-cells (CD8+) to regulatory T-cells ratio was associated with a higher risk of rejection and reduced likelihood of graft tolerance(85, 86). |
| | Liver transplant (24) | Prospective cohort | Pre-transplant T-cell dysregulation with increased proportion of terminally differentiated memory T-cells (TEMRA) and lower naiive T-cell proportion associated with rejection(87). |
| | Kidney (185) | Prospective cohort | Increase in differentiated T-cell (CD28-CD8+) associated with rejection. CD28-CD8+ exhibit stronger T-cell receptor and FcγRIIIA (CD16) interactions (increase in IFNγ, TNFα, and CD107a expression)(88). |
| | Kidney (1032) | Prospective cohort | Reduction in peripheral blood naiive T-cells (CCR7+CD8+) and an increase in effector T-cell profile (CD28-CD57+CD8+ and CCR7-CD45RA+CD8+ (TEMRA)) was strongly associated with allograft rejection(87-89). |
| **NK-cell** | Kidney transplant (168) | Prospective cohort | Impaired NK cell function, not number, significantly associated with risk of infection.(90) Lower NK (CD56+, CD16+) at 1-month post-transplant associated with higher risk of OI. |
| | Liver transplant (92) | Prospective cohort | Lower NK cell count (<0.050 x $10^3$ cells/ μL) was the strongest predictor of CMV disease and other OI(91). |
| | Liver transplant (35) | Prospective cohort | Corticosteroid had the greatest impact on NK number and function, followed by CNI and MMF. mTOR inhibitors had the least impact on NK cell cytolytic function(92). |
| | Kidney (mouse model) | Prospective observational | NK and DSA interaction required for AMR. DSA alone without NK leads to chronic damage without rejection(93). NK implicated in vascular lesions associated with DSA mediated injury. |
| | Kidney (25) | Case-control Study | KTRs with chronic AMR (cAMR) had a higher percentage of monocytes and an increase in activated NK cell phenotype (CD16+) compared to the no-cAMR group(94). |
| | Lung (41) | Prospective observational | Lung transplant recipients with chronic rejection had a decrease in total peripheral NK cell number but an increase in activated peripheral NK cell number (CD16) and increase in total |

| | | | |
|---|---|---|---|
| | | | intragraft NK cell number, ? due to migration into graft during rejection (95). |
| | Liver (32) | Prospective observational | Rejection associated with higher immature (CD56+$^{bright}$) and activated NK cell (high NKp30 expression) phenotypes (96).<br>Elevated immature (CD56+$^{bright}$) NK cells and reduced peripheral mature CD56+$^{dim}$ (Intragraft homing) and increased Intragraft CD56+$^{dim}$ associated with AMR(76). |
| **Immunoglobulins Hypogamma-globulinaemia (HGG)** | Kidney (226) | Prospective cohort | HGG was common and most profound at 1 month (52%) post kidney transplant and 31% had persistent HGG at 6 months post-transplant(97).<br>HGG at 1- and 6-months post-transplant increased risk of infections in the 1-6months and >6month periods respectively(97). HGG associated with 2.3x fold higher risk of infections(97). |
| | All SOT (1756) | Meta-analysis | Severe HGG (IgG<400mg/dL) in the first year significantly increased risk of CMV, fungal and respiratory infections and increased 1-year all-cause mortality 21.91x fold(98). |
| | Kidney (192) | Retrospective cohort | Early HGG associated with high risk of infection requiring hospitalisation and graft viral infections(99).<br>Intravenous immunoglobulin therapy in hypogammaglobulinaemic SOT recipients, reduces the rates and severity of re-infections(37, 100, 101). |
| **Complements** | Kidney (270) | Prospective cohort | 1-month C3 hypocomplementaemia (↓C3) increased the intermediate (1-6month) risk of overall, bacterial and fungal infections and 6-month ↓C3 increased risk of bacterial infections beyond 6 months(102) |
| | Liver (46) | Prospective cohort | Pre-transplant C3 hypocomplementemia associated with increased risk of infections(103). |
| **Mannose Binding Lectin (MBL)** | Liver (240) | Prospective cohort | MBL deficient liver transplant had higher incidence of overall and bacterial infections, shock and pneumonia and increased 1-year bacterial infection associated mortality(104). |
| **Soluble CD30** ***Transmembrane glycoprotein of the*** | Heart (100) | Retrospective cohort | High pre-transplant sCD30 (>90U/mL) associated with more robust immune response and reduced risk of infection post-transplant(105). |

| | | | |
|---|---|---|---|
| ***tumor necrosis factor (TNF) family.*** *Cleaved from activated effector and memory T-cells* | Kidney (100) | Prospective cohort | Higher sCD30 (≥13.5ng/mL) at transplant associated with ↑ risk of bacterial infection across 12 months,? due to Th2-polarized T-cell response and impaired bacteria specific immunity(106). |
| | Kidney (100) | Retrospective cohort | Low pre-transplant sCD30 (120U/ml) associated with higher risk of pneumonia(107). |
| | Kidney (620) | Prospective observational | Higher sCD30 levels 1-year post-transplant correlated with higher risk of graft loss in the subsequent 5 years. Effects were additive in those who also developed denovo DSA(108). |
| | SOT (1453) | Meta-analysis | Strong association between elevated post-transplant sCD30 levels and acute allograft rejection. The association was more pronounced in deceased donor transplants (74). |
| | SOT (2507) | Meta-analysis | Pre-transplant sCD30 showed poor accuracy for predicting allograft rejection(109). |
| | Kidney (73) | Prospective cohort | sCD30 sample at 7-14 days post-transplant was the best time to predict rejection. Serial pre-and post-transplant measurements were more informative of rejection risk than a single measurement alone(110). |
| **iATP (ImmuKnow Assay)** ***Stimuli induced iATP assessment.*** *Describes T-cell activation and function/ immunocompetence* | Heart (80) | Prospective cohort | ImmuKnow assay-based temporary IS reduction allowed for resolution of the severe infection, and normalization of ImmuKnow assay levels, without any episodes of rejection. Maintenance IS recommenced when ImmuKnow assay level reached >225ng/mL of ATP(111). |
| | Kidney (71) | Prospective cohort | Serial iATP measurements before, during and after BK infection showed significant temporal differences. iATP increased from pre-infection through to the pre-recovery phase post-BK viremia(112). |
| | Heart (50) | Prospective cohort | Median ImmuKnow assay levels were lower in those who had developed infection compared to those who did not (129ng ATP/mL vs. 351ng ATP/mL, $p<0.05$)(113). |
| | Heart (50) | Prospective cohort | Median ImmunKnow assay levels were higher in those who had developed rejection compared to those who did not (619 ng ATP/mL vs 351ng ATP/ml, $p<0.05$)(113). |

| | |
|---|---|
| | Most studies on the utility of iATP in infection/rejection risk prediction is heterogeneous, and the evidence is particularly unclear and discordant with respect to its ability to identify allograft rejection(75, 114, 115). |

CMV, cytomegalovirus; KTR, kidney transplant recipient; OI, opportunistic infections; SOT, solid organ transplant; FcγRIIIA, Fc region receptor IIIA; IFNγ, interferon gamma; TNFα, tumor necrosis factor alpha; NK, natural killer; ; CNI, calcineurin inhibitor; MMF, mycophenolate; mTOR, mammalian target of rapamycin; DSA, donor specific antibody; AMR, antibody mediated rejection; C3, complement component 3; Th2, T-helper2; iATP, intracellular adenosine triphosphate
